# Climate change mitigation in British Columbia’s forest sector: GHG reductions, costs, and environmental impacts

**DOI:** 10.1186/s13021-020-00155-2

**Published:** 2020-10-01

**Authors:** C. E. Smyth, Z. Xu, T. C. Lemprière, W. A. Kurz

**Affiliations:** 1 Natural Resources Canada, Canadian Forest Service, 506 Burnside Road West, Victoria, BC V8Z 1M5 Canada; 2 Natural Resources Canada, Canadian Forest Service, 580 Booth Street, Ottawa, ON K1A 0E4 Canada; 3 Natural Resources Canada, Canadian Forest Service, 300-655 Bay St, Toronto, ON M5G 2K4 Canada

**Keywords:** Climate change mitigation, Forest sector, British Columbia, Cost per tonne, Socio-economic impact, GCBM

## Abstract

**Background:**

The potential contributions from forest-based greenhouse gas (GHG) mitigation actions need to be quantified to develop pathways towards net negative emissions. Here we present results from a comparative analysis that examined mitigation options for British Columbia’s forest sector. Mitigation scenarios were evaluated using a systems perspective that takes into account the changes in emissions and removals in forest ecosystems, in harvested wood product (HWP) carbon stocks, and in other sectors where wood products substitute for emission-intensive materials and fossil fuels. All mitigation activities were assessed relative to a forward-looking ‘business as usual’ baseline for three implementation levels. In addition to quantifying net GHG emission reductions, we assessed economic, and socio-economic impacts as well as other environmental indicators relating to forest species, age class, deadwood availability and future timber supply. We further considered risks of reversal for land-based scenarios, by assessing impacts of increasing future wildfires on stands that were not harvested.

**Results:**

Our spatially explicit analyses of forest sector mitigation options demonstrated a cost-effective portfolio of regionally differentiated scenarios that directed more of the harvested wood to longer-lived wood products, stopped burning of harvest residues and instead produced bioenergy to displace fossil fuel burning, and reduced harvest levels in regions with low disturbance rates. Domestically, net GHG emissions were reduced by an average of -9 MtCO_2_e year^−1^ over 2020–2050 for a portfolio of mitigation activities at a default implementation level, with about 85% of the GHG emission reductions achieved below a cost of $50/tCO_2_e. Normalizing the net GHG reduction by changes in harvested wood levels permitted comparisons of the scenarios with different ambition levels, and showed that a 1 MtCO_2_ increase in cumulative harvested stemwood results in a 1 MtCO_2_e reduction in cumulative emissions, relative to the baseline, for the *Higher Recovery* scenario in 2070.

**Conclusions:**

The analyses conducted in this study contribute to the global understanding of forest sector mitigation options by providing an integrated framework to synthesize the methods, assumptions, datasets and models needed to quantify mitigation activities using a systems approach. An understanding of economically feasible and socio-economically attractive mitigation scenarios along with trade offs for environmental indicators relating to species composition and age, helps decision makers with long-term planning for land sector contributions to GHG emission reduction efforts, and provides valuable information for stakeholder consultations.

## Background

According to future emissions scenarios, keeping the global average temperature increases to well below two degrees Celsius above pre-industrial levels requires negative net greenhouse gas (GHG) emissions through the end of this century [26]. The landmark agreement to combat climate change that was reached in Paris in 2015 [59] aims to achieve net zero emissions in second half of this century (Article 4), and includes commitments to enhance and conserve forest-based carbon (C) sinks (Article 5). Canada has committed to a 30% reduction in emissions by 2030 (relative to 2005 emissions) [9], and to contribute to this reduction the Pan-Canadian Framework for Clean Growth and Climate Change provides financial incentives for rehabilitation of forests after natural disturbances, construction of innovative wood structures, and the use of wood for heating in remote and rural communities in place fossil fuel burning [10]. British Columbia (BC), the region of interest in this study, has committed to reducing BC’s GHG emissions by 80% by 2050 (relative to 2007 emissions) and the CleanBC plan includes recovering more wood fibre, and avoiding emissions from burning post-harvest residuals [16].

Determination of the GHG reduction and associated costs of forest management and forest-derived products is complex, and a comprehensive integrated analysis is needed to support policy initiatives by quantifying emissions and removals in the forest ecosystem, tracking emissions from harvested wood products including bioenergy, and considering emissions in the interacting energy and industrial products sectors [38, 43]. Management of forests and harvested wood products has been shown to have substantial global potential to mitigate climate change by reducing greenhouse gas (GHG) emissions or enhancing carbon sequestration [38], and through the use of wood products to displace emissions-intensive materials and fossil fuels [18, 65]. In Canada, GHG emissions reduction studies have found forest-related strategies may be cost-effective choices to help achieve long-term emission reductions at the national level [32, 49] and at the provincial level for British Columbia [67].

In addition to GHG emissions reductions and costs, forest management strategies can impact the area of old forests and deadwood availability, which can affect biodiversity, and wildfire risk. These and other variables influence the level of public support for forest management strategies [44] and the effectiveness of resource management policies, which depends on the general level of understanding, acceptance, and perception of them as being effective, fair and legitimate [27, 51]. In Finnish boreal forests, increasing harvest levels increased timber production, but decreased the total system C balance and reduced the area of old forests and dead wood, which could negatively impact biodiversity [20]. Harvesting in Canadian boreal forests was found to affect large-animal predation rates, and bird, caribou, and small mammal communities by changing the forest species composition, creating a younger age-class distribution, and reducing deadwood [61].

Our objectives were to examine the biophysical climate change mitigation potential for six mitigation scenarios (Table 1), assess mitigation costs and socio-economic impacts, and summarize impacts of these mitigation activities on environmental indicators related to forest species distribution, age-class distribution, future timber supply, and available deadwood. We examined forest management scenarios that increased stand-level C density through reductions in harvest, or used harvest residues for energy production and reduced slash-pile burning, or reduced waste by using more of the harvested wood for wood products. We further examined a harvested wood products (HWP) scenario that shifted wood commodities towards longer-lived products, and combined this with forest management scenarios. Emissions from the forest ecosystem and harvested wood products were considered at various scenario implementation levels, along with a range of substitution benefits of using bioenergy in place of contemporary and future fossil fuel energy, and solid wood products in place of alternates such as plastic, steel, and concrete.

**Table 1:** Description of GHG reduction scenarios, three implementation levels, and high and low substitution benefits, as well as a description of the *baseline*

Forest management and wood use scenarios			
Scenario	Description	Implementation levels	Substitution benefits
Harvest less	Harvest area is reduced, relative to the *baseline*. Harvest areas are randomly selected and removed from the harvest schedule. Wildfire risk of conserved stands is considered *ex*-*post*	10% harvest reduction Low: 2% High: 20%	Solid wood product benefits are based on the change in sawnwood and panels, relative to the *baseline* levels. High benefits assumed that the incremental wood is used in construction, low benefits assumed that wood is used in a wide range of products
Restricted harvest	Harvest area of old stands is reduced, relative to the *baseline*. Old is defined by natural disturbance type time interval. Wildfire risk of conserved stands is considered ex-post	Reduced harvest of old stands, where the old age threshold varies between 150 and 250 years, depending on the region Low: 175 to 300 years High: 125 to 200 years	As above
Higher recovery	Increase the use of harvested stemwood for wood products, without changing the harvest area	5% increase from the *Baseline* level of 85% stemwood C in merchantable-sized trees is used for products (in all but four regions) Low: 3% increase High: 8% increase	As above
Residues for bioenergy	Collect a portion of harvest residues (including branches, small trees, tops, and stemwood from unused merchantable-sized trees and snags), stop slashpile burning, and generate bioenergy (heat, and/or power)	25% of harvest residues are collected for bioenergy Low: 20% High: 30%	Bioenergy benefits were based on substitution of fossil fuel burning with high benefits for contemporary fossil fuel use, and low benefits for lower fossil fuel use in the future
Higher recovery and residues for bioenergy	Combination of two scenarios, where (1) the use of harvested stemwood for products is increased and (2) a portion of harvest residues is collected for bioenergy and slashpile burning is stopped	5% increase in stemwood utilization and 25% of harvest residues collected Low: 3% increase in utilization, 20% of residues collected High: 8% increase in utilization, 30% of residues collected	As above for solid wood products
Longer-lived wood products	This wood use scenario can be combined with all forest management scenarios listed above. Wood products are shifted immediately from pulp and paper towards longer-lived sawnwood and panels. Mill residues are unchanged	Wood products are 55% sawnwood, 22% panels, and 21% pulp and paper Low: 53%, 20%, 24% (respectively) High: 56%, 23% and 18% (respectively)	As above for solid wood products
*Baseline* description			
The *baseline* modeled carbon stocks and GHG emissions and removals at 1-ha resolution from 1990 to 2070 for 62.9 Mha of public forests in British Columbia. Future harvest and wildfires were projected, with no interaction between wildfires and conserved stands. Projections of harvesting were based on contemporary practices of harvest utilization, slashpile burning of harvest residuals, and bioenergy production from mill residuals. Wood product commodities (sawnwood, panels, pulp and paper, and other industrial roundwood) were based on contemporary levels with an assumed downward trend in pulp and paper production. Each commodity had an assumed half-life, after which commodities were sent to landfills, incinerated or used for energy			

We build upon previous research which assessed the climate change mitigation potential and economic feasibility Smyth et al. [49, 48], Xu et al. [67] by: expanding the analyses to use multiple scenario implementation levels; including additional environmental indicators; normalizing GHG reductions to enable scenario comparisons with different implementation levels; and including the risk of wildfires for conserved stands (*ex*-*post*). Earlier methods have also been improved by using spatially explicit forest C modeling for a longer (50 year) time period, and economic assumptions have been refined and updated.

## Results

### Climate change mitigation potential

**Fig. 1:**
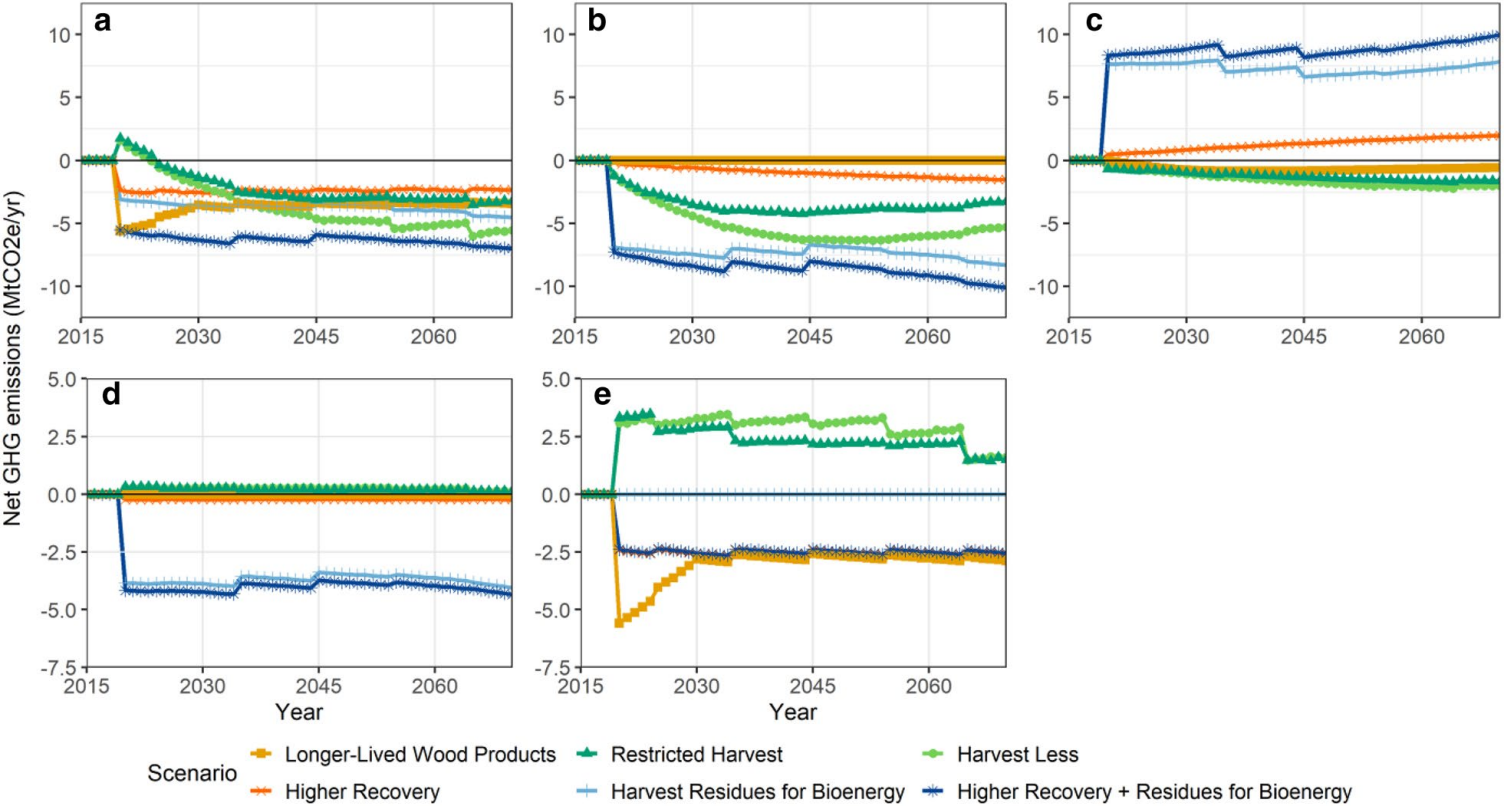
Timeseries of changes in net greenhouse gas (GHG) emissions and removals components for the default scenario implementation level and high substitution benefits. **a** Total, **b** Forest ecosystem, **c** harvested wood products emissions, **d** energy substitution benefits from avoided contemporary community and industrial energy uses and **e** product substitution benefits assuming incremental wood use in buildings. Enhanced removals or reduced GHG emissions for mitigation activities (relative to the *baseline*) are denoted by negative values. Note that panels d and e have a smaller y-axis range

Figure 1 shows the timeseries of the total annual mitigation potential and its components (forest ecosystem, HWP emissions, and substitution benefits from energy and products) for the default scenario implementation level. Scenarios involving the collection of residues for bioenergy, *Harvest Residues for Bioenergy* and *Higher Recovery and Residues for Bioenergy* have the greatest mitigation in the forest ecosystem (Fig. 1b) because C in residues that would have been slashpile burned or left to decay in the *baseline* scenario were transferred out of the forest, resulting in a reduction in emissions compared to the *baseline*. Emissions from C used for bioenergy and wood products are captured in the HWP component (Fig. 1c) where these two scenarios show a large increase in emissions relative to the *baseline*. The use of wood for bioenergy avoids fossil fuel burning (Fig. 1d) where regionally differentiated bioenergy facilities and avoided fossil fuel emissions were determined by the available biomass supply and fossil fuel energy demand within each of the 38 regions.

The use of wood to substitute other materials provided substitution benefits (Fig. 1e) for the *Higher Recovery* and *Higher Recovery and Residues for Bioenergy* scenarios, where incremental wood products (relative to *baseline* levels) was assumed to replace steel and concrete in buildings in the high substitution benefits assumption. The *Higher Recovery* scenario had reduced emissions in the forest ecosystem, relative to the *baseline*, because C which would have been slashburned or left to decay was transferred to wood products. However, HWP emissions are larger than the *baseline* for this scenario because of the incremental C transferred to HWP.

The *Longer*-*Lived Products* wood use scenario did not alter emissions in forest ecosystems because harvest levels were the same as the baseline but it had reduced HWP emissions due to delayed post-consumer emissions, and higher substitution benefits associated with incremental production of sawnwood and panels, relative to the *baseline*. Substitution benefits were larger in the first decade because we assumed longer-lived products were produced immediately, whereas the *baseline* had a slow increase in longer-lived products from 2015 to 2030.

The two conservation scenarios which involved reduced harvest levels, *Harvest Less*, and *Restricted Harvest* had fewer ecosystem emissions because fewer stands were harvested and conserved stands continued as forest sinks. However, the mitigation component of the forest ecosystem reached a maximum after a few decades and then decreased because of regrowth of post-harvested stands in the *baseline*, and a loss of mitigation potential associated with conserved stands that were burned in wildfires. Risk of reversal from wildfires was considered *ex*-*post* for conservation scenarios based on the interaction between conserved stands and statistically-based future wildfires. Including the average risk reversal decreased the cumulative mitigation potential by 12% in 2070 for the southern interior, a reduction of 15% in the northern interior, and 3% in the coastal regions (Additional file 1: Table S8). These modest reductions in the cumulative mitigation reflect small (< 1%) average annual interaction levels between wildfires and conserved stands. However, burned areas have a high uncertainty, and the uncertainty range in the area burned based on the 95% confidence interval range [37] was ~ 2.5% (averaged over 50 years) (Additional file 1: Figure S6).

Conservation scenarios had reduced emissions from HWP (Fig. 1c), but incurred fewer substitution benefits from industrial bioenergy associated with mill residues, and fewer substitution benefits from products (Fig. 1e), relative to the *baseline*. For the scenarios that have lower harvest levels, the consequence of fewer substitution benefits is that it diminishes their overall effectiveness at reducing GHG emissions.

**Fig. 2:**
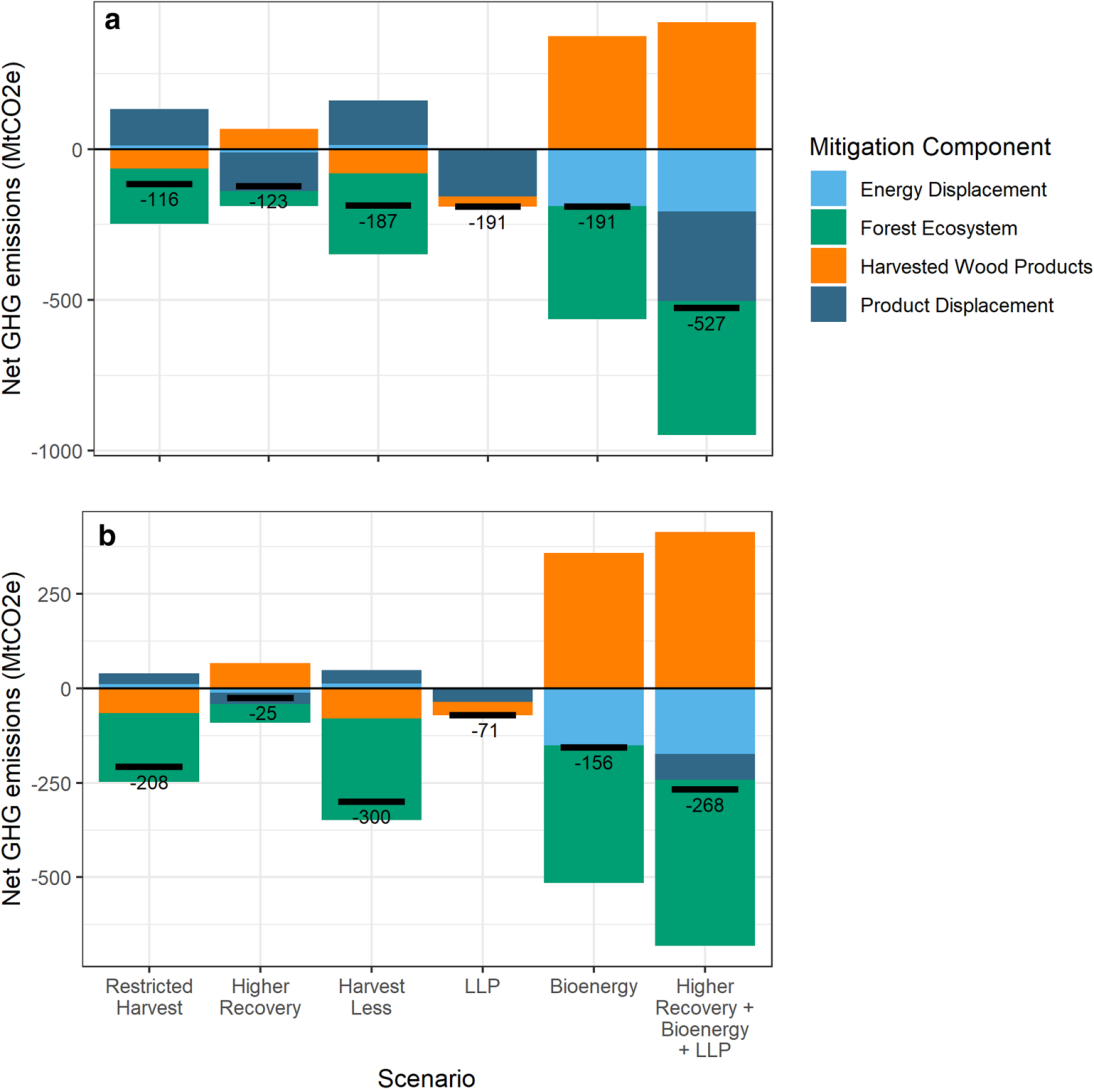
Cumulative mitigation in 2070 at a default implementation level for **a** high and **b** low substitution benefits. Total cumulative mitigation is indicated by the black horizontal line, and mitigation components for the forest ecosystem, harvested wood products and substitution components are indicated by coloured bars. LLP stands for Longer-Lived Products

The total cumulative mitigation potential in 2070 and contributions from each of the components is shown in Fig. 2. There are many combinations of activities that could be explored, but because activities typically change the harvest level or the flow of biomass, combinations of activities must be modeled together and cannot be added *ex*-*post*. We modeled a combination of forest management scenarios where more of the harvested wood was directed to products, a greater share of products were longer-lived wood products, and a portion of harvest residues was collected for bioenergy. This combination of activities had the highest mitigation potential for the default implementation level and high substitution benefits assumption.

**Fig. 3:**
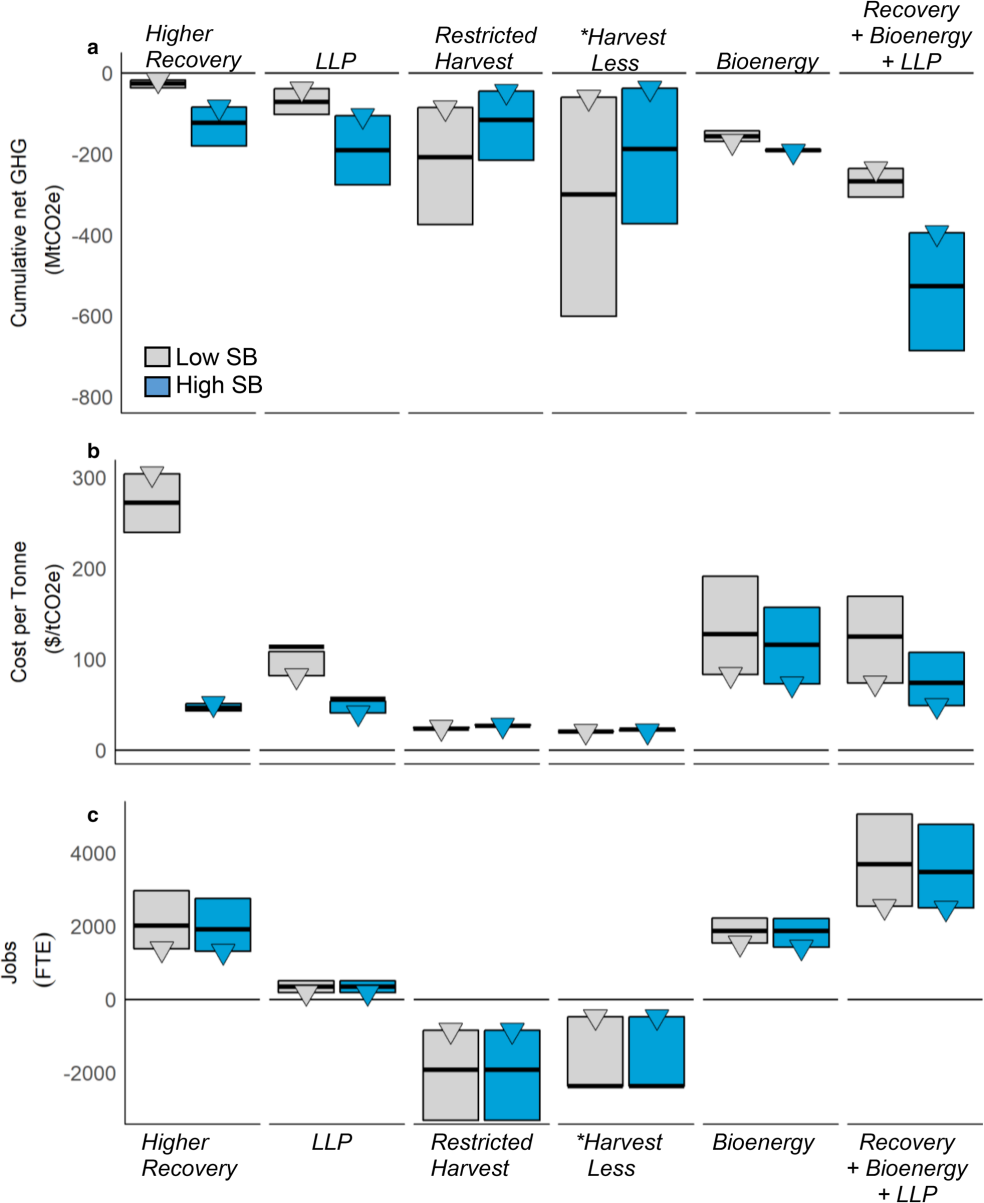
**a** Cumulative emissions reduction (global) for mitigation strategies (2020–2070), **b** overall average cost per tonne of emissions reduction (domestic), and **c** changes in the number of jobs within and connected to the forest sector within Canada. Bars indicate the range of the three implementation levels (low—triangle symbol, default—thick black line, and high—black line). Colours indicate two levels of substitution benefits (SB) (low—gray and high—blue). LLP stands for Longer-Lived Products. Asterisk estimate of costs and jobs are not available for the high implementation level for the *Harvest Less* scenario

The results presented thus far have been based on a default implementation level of mitigation activities, and high substitution benefits from avoiding contemporary fossil fuels and using incremental wood to substitute steel and concrete in construction. In order to assess the impacts of varying implementation levels and substitution benefits, we repeated the comparative analyses of mitigation potential for high and low implementation levels, and included low substitution benefits from avoiding future energy fuels and using incremental wood for general uses. Figure 3a shows the cumulative mitigation potential in 2070 for all scenarios, three implementation levels (high, default, low) and two levels of substitution benefits for both energy and products (see also Additional file 1: Table S7 for additional scenario combinations). Generally, a higher implementation level increased the mitigation potential, with the exception of scenarios involving bioenergy. The *Harvest Less* scenario had the largest range in mitigation potential, because of the large range in the implementation levels. Harvest areas were reduced by 2%, 10% and 20% relative to the *baseline*, for the low, default and high implementation levels, respectively, which resulted in harvest volume reductions of 1.8%, 7.6%, and 18.4%. Harvest reductions for the *Restricted Harvest* scenario were within a smaller range, with reduced harvest volumes for the three implementation levels of 3.2%, 5.6% and 12.6%.

Using incremental wood in buildings had higher substitution benefits than general use, and increased the mitigation potential of the *Higher Recovery* and *Longer*-*Lived Products* scenarios, but had the opposite effect on conservation scenarios where reduced substitution benefits act as a penalty.

For the *Bioenergy from Harvest Residues* scenario, varying the collection rates from 20% for low, 25% for default and 30% resulted in collected residues of 3.4 Mm^3^ year^−1^, 4.2 Mm^3^ year^−1^, and 5.1 Mm^3^ year^−1^ respectively. Average avoided emissions for these implementation levels were 0.50, 0.46 and 0.44 tC avoided per tC used for bioenergy, indicating that at the provincial level, the substitution benefit per unit of tC collected decreased with additional biomass because it was directed towards electricity production which avoided low emissions grid electricity (Additional file 1: Figure S3). In up to five regions (depending on the implementation level) the use of harvest residues for bioenergy increased the net GHG emissions, because bioheat production exceeded local heat demand and excess biomass was consequently used to avoid low emissions electricity. Substitution benefits from future energy fuels were found to be smaller than those from contemporary fuels because future fuels had lower emissions intensities (Additional file 1: Table S4, and Additional file 2), resulting in smaller substitution benefits (Additional file 1: Figure S3).

Portfolios were constructed by selecting the best combination of scenarios (Additional file 1: Figure S4) in each region for two goals (maximize the global (defined as within BC and elsewhere) cumulative mitigation, or maximize the domestic (within BC) cumulative mitigation), over three time periods (2020–2030, 2020–2050 or 2020–2070). The annual average mitigation potential for these portfolios was − 10 to − 11 MtCO_2_e year^−1^ for global portfolios, resulting in a cumulative mitigation potential of − 539 MtCO_2_e year^−1^ in 2070 (Table 2). Annual average domestic mitigation potential was about 10% to 40% less depending on the decade and portfolio, resulting in a cumulative mitigation potential of − 428 MtCO_2_e year^−1^ in 2070. Changing the scenario implementation level resulted in a range of global mitigation of − 400 MtCO_2_e year^−1^ and − 736 MtCO_2_e year^−1^, for low and high implementation levels, respectively (Additional file 1: Table S6).

**Table 2:** Annual average mitigation potential (MtCO_2_e year^−1^) for portfolios by decade for default scenario implementation levels and high substitution benefits (SB)

Decade	Short-term 2030 portfolio		Mid-term 2050 portfolio		Long-term 2070 portfolio	
	Global	Domestic	Global	Domestic	Global	Domestic
2020–2029	− 11.1	− 7.2	− 11.0	− 7.0	− 10.5	− 6.4
2030–2039	− 10.2	− 7.7	− 10.2	− 8.2	− 10.3	− 8.3
2040–2049	− 10.0	− 8.0	− 10.1	− 8.8	− 10.3	− 9.1
2050–2059	− 10.0	− 8.0	− 10.2	− 8.9	− 10.6	− 9.4
2060–2069	− 10.4	− 8.2	− 10.6	− 8.9	− 11.1	− 9.3
Total	− 529	− 396	− 533	− 425	− 539	− 428

**Fig. 4:**
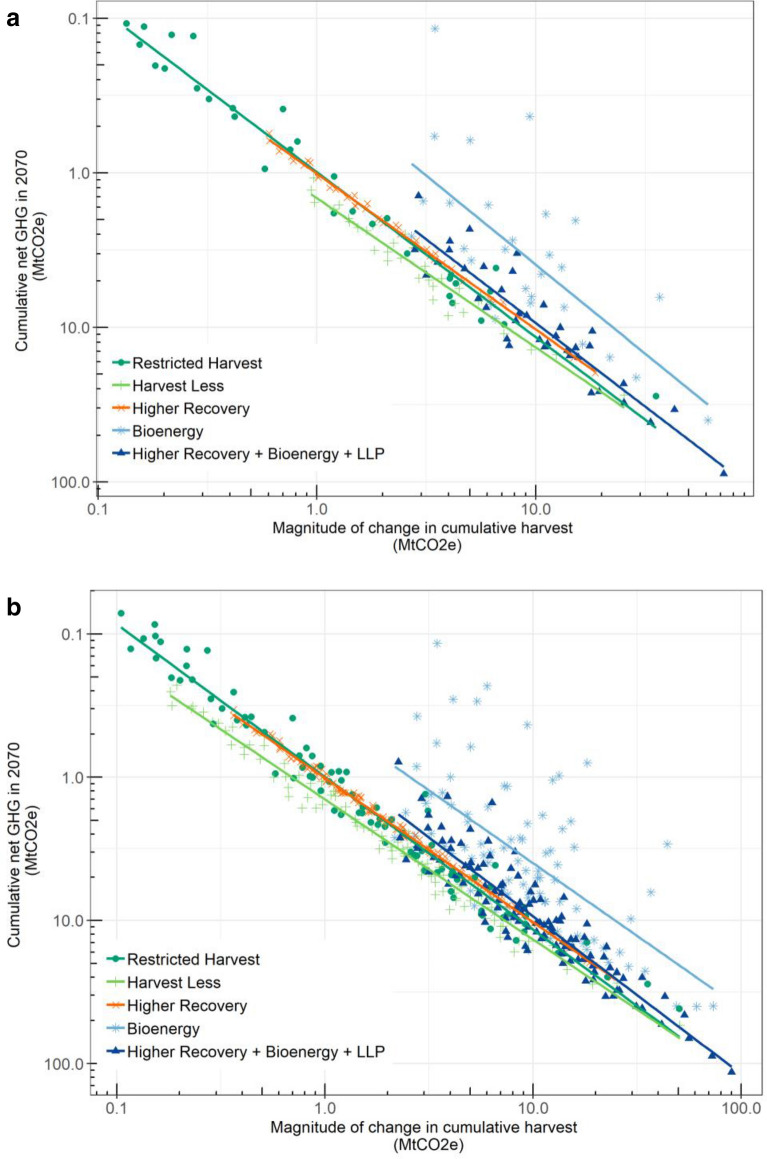
Cumulative net GHG emissions in 2070 compared to the magnitude of the associated cumulative change in harvest C, relative to the *baseline,* for each region (points) along with linear regressions (lines) for **a** default scenario implementation level and **b** all implementation levels, assuming high substitution benefits. Small cumulative net emissions (smaller than − 0.1 MtCO_2_e) have been excluded. LLP stands for Longer-Lived Products

Portfolios selected the best mix of regionally differentiated scenarios for each of the three implementation levels, but these levels were developed independently for each scenario and their different ranges may affect their ranking. It is advantageous to generalize the existing results so that we can estimate the net change in GHG emissions for any implementation level within the modeled range. Figure 4a shows the 2070 cumulative mitigation potential (default implementation level, high substitution benefits) for each region plotted against the absolute value of the cumulative change in harvested wood (including roundwood and residues) relative to the baseline, and although the regions differed in size and harvesting activity, there was a well-defined relationship for most scenarios. Including all of the implementation levels (Fig. 4b, Additional file 1: Table S7) resulted in very similar regressions, indicating the cumulative mitigation potential could be estimated from the change in harvested wood (relative to the baseline). Slopes from the log–log regressions were close to -1 for the *Higher Recovery* scenario (between − 0.5 and − 1.2 for other scenarios), indicating a 1 MtCO_2_ increase in cumulative harvested wood in 2070 resulted in a change (relative to the baseline) of − 1 MtCO_2_e in cumulative emissions in 2070. The *Bioenergy* scenario had the greatest variation amongst the regions, which was caused by the degree to which available biomass for bioenergy could meet the local heat demand and substitute high-emissions fossil fuels (See Additional file 2). Normalized net GHG reductions, defined as the net change in cumulative GHG emissions divided by the cumulative change in harvested wood for the *Higher Recovery* scenario were − 1 for all implementation levels in most regions, while other scenarios had more regional variability (Additional file 1: Figure S5). For the conservation scenarios, the normalized net GHG reduction was greater for the *Harvest Less* scenario than for the *Restricted Harvest* scenario in most regions, indicating that, of the two conservation scenarios, the *Harvest Less* scenario would have a greater mitigation benefit.

### Economic and socio-economic analyses

Table 3 summarizes the provincial annual average cost impacts for the entire period for all scenarios and the domestic portfolio under the default scenario implementation level. Costs for all implementation levels are shown in Fig. 3b and given in Additional file 1: Table S18.

**Table 3:** Average cost per tonne estimates by scenario for default level of implementation ($ tCO_2_e^−1^ in 2018 Canadian dollars), 2020–2070

Scenario	Low substitution benefits		High substitution benefits	
	General use	Future fuels	Wood buildings	Contemporary fuels
Higher recovery	272		47	
Harvest less	22		25	
Bioenergy		126		114
Restricted harvest	24		29	
Longer-lived products	114		56	
Higher recovery + bioenergy	113		94	
Higher recovery + bioenergy + LLP	127		88	
Domestic portfolio	29		34	

In terms of individual scenarios, the *Restricted Harvest* and *Harvest Less* scenarios have the lowest mitigation costs ($20–$30 per tCO_2_e), but in terms of socio-economic impacts, there were significant reductions in jobs (Fig. 3c), Gross Domestic Product (GDP) and government revenue (Table 4, Additional file 1: Table S19). The *Harvest Residues for Bioenergy*, *Higher Recovery plus Harvest Residues for Bioenergy,* and *Longer*-*Lived Products* (*LLP)* scenarios indicated moderate mitigation costs ($94–$126 per tCO_2_e). The *Higher Recovery* scenario with low substitution benefits had positive socio-economic impacts, but indicated the highest mitigation cost ($272 per tCO_2_e) due to limited mitigation potential. The *Higher Recovery* scenario had the greatest cost per tonne difference between the low and high substitution benefits, reflecting the significant difference in mitigation potentials depending on how the incremental harvest was used.

**Table 4:** Socio-economic impacts by scenario for default level of implementation, 2020–2070

Scenario	Forest job	Total job	Forest GDP	Total GDP	Gov. revenue
	(Total FTE)	(Total FTE)	(2018$M year^−1^)	(2018$M year^−1^)	(2018$M year^−1^)
Higher recovery (general use)	947	1921	68	132	9
Higher recovery (wood buildings)	993	2015	71	138	9
Harvest less	− 1167	− 2362	− 102	− 196	− 14
Bioenergy (contemporary fuels)	1040	1880	315	363	48
Bioenergy (future fuels)	1040	1880	299	345	45
Higher recovery + bioenergy + LLP (low SB)	2091	3688	286	318	40
Higher recovery + bioenergy + LLP (high SB)	2170	3830	303	338	43
Higher recovery + bioenergy (low SB)	1649	3130	319	405	48
Higher recovery + bioenergy (High SB)	1760	3339	341	432	51
Restricted harvest	− 945	− 1912	− 86	− 164	− 11
LLP	333	351	− 46	− 103	− 9
Domestic portfolio (Low SB)	− 1019	− 2343	− 156	− 313	− 24
Domestic portfolio (High SB)	− 177	− 714	− 29	− 135	− 5

*FTE* full time equivalent, *GDP* gross domestic product, *Gov.* government, *LLP* longer-lived product, *SB* substitution benefits

Scenarios involving bioenergy had very high socio-economic impacts because bioenergy production from harvest residues was a new industry and generated substantial revenue.

Changing the scenario implementation level had little impact on the cost per tonnes for the conservation scenarios, due to the proportional changes in total cost and cumulative mitigation, but it greatly affected the cost per tonne in bioenergy scenarios because changing the level of collected harvest residues affected bioenergy facility selection and avoided fossil fuels. Except for conservation scenarios, each scenario increased jobs, but the *LLP* scenario resulted in losses in GDP and government revenue because the pulp and paper industry is more capital intensive and less labour intensive as compared to wood manufacturing. The cost per tonne values for domestic portfolios are among the lowest, with minimal variations between implementation levels and substitution benefits (Additional file 1: Table S18).

**Fig. 5:**
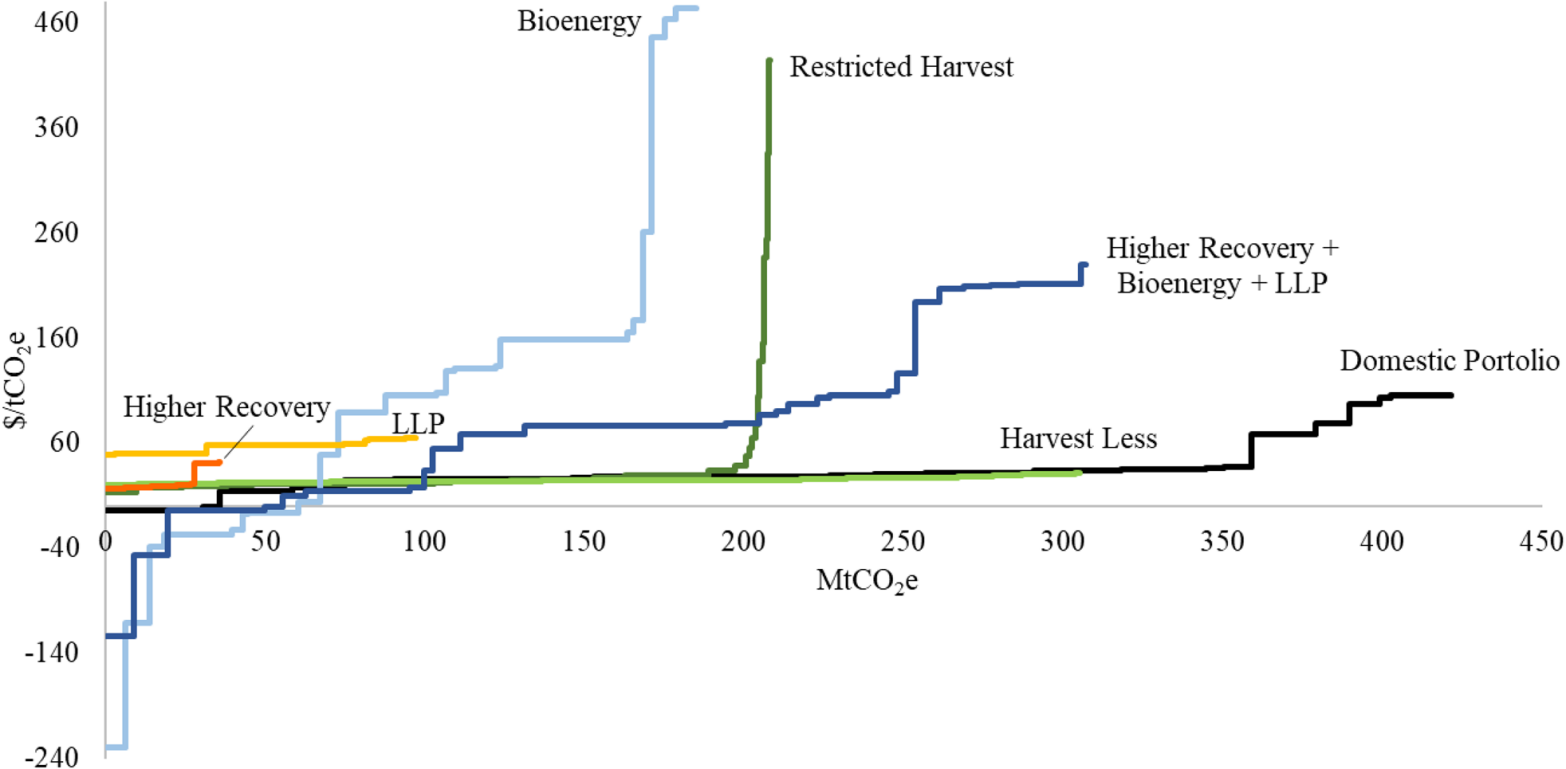
Cost curves for domestic mitigation for individual scenarios and the domestic portfolio with high substitution benefits (wood in buildings and contemporary fuels) and default implementation level, 2020–2070. Some extreme values have been eliminated for display purposes. LLP stands for Longer-Lived Products

In addition to the average costs presented so far, cost curves showing the regional cost per tonne values for domestic mitigation for the default scenario implementation level and high substitution benefits are shown in Fig. 5. Cost curves were constructed by ranking cost per tonne values from the lowest to the highest and plotting them against the cumulative mitigation potential. Cost curves for *Harvest Less*, *LLP*, and *Restricted Harvest* scenarios were relatively flat, with similar costs per tonne for most regions. The *Higher Recovery* scenario indicated a limited domestic mitigation potential with very regionally differentiated costs (steep slope). The *Bioenergy* and *Higher Recovery and Bioenergy* scenarios had large regional variability because different numbers and types of bioenergy facilities were selected to substitute different fossil fuels by the optimization model based on local energy demands and harvest residue availability, transportation distances (simplified), and production costs for both bioenergy and fossil fuel energy being displaced. Most mitigation benefits came from substituting bioenergy for heat and power generated using natural gas, fuel oil and diesel, with the shares of these fossil fuels in total energy consumption varying by region (See Additional file 2). For populated regions with large energy demands, high costs often occurred because natural gas was substituted, which is generally much cheaper than bioenergy. The cost curve for the domestic portfolio demonstrates the most cost effective pathway among scenarios to achieve the highest cumulative mitigation by 2070. The variations of cost curves for the domestic portfolio for default and low implementation levels and high and low substitution benefits are shown in Additional file 1: Figure S9.

### Other indicators

**Fig. 6:**
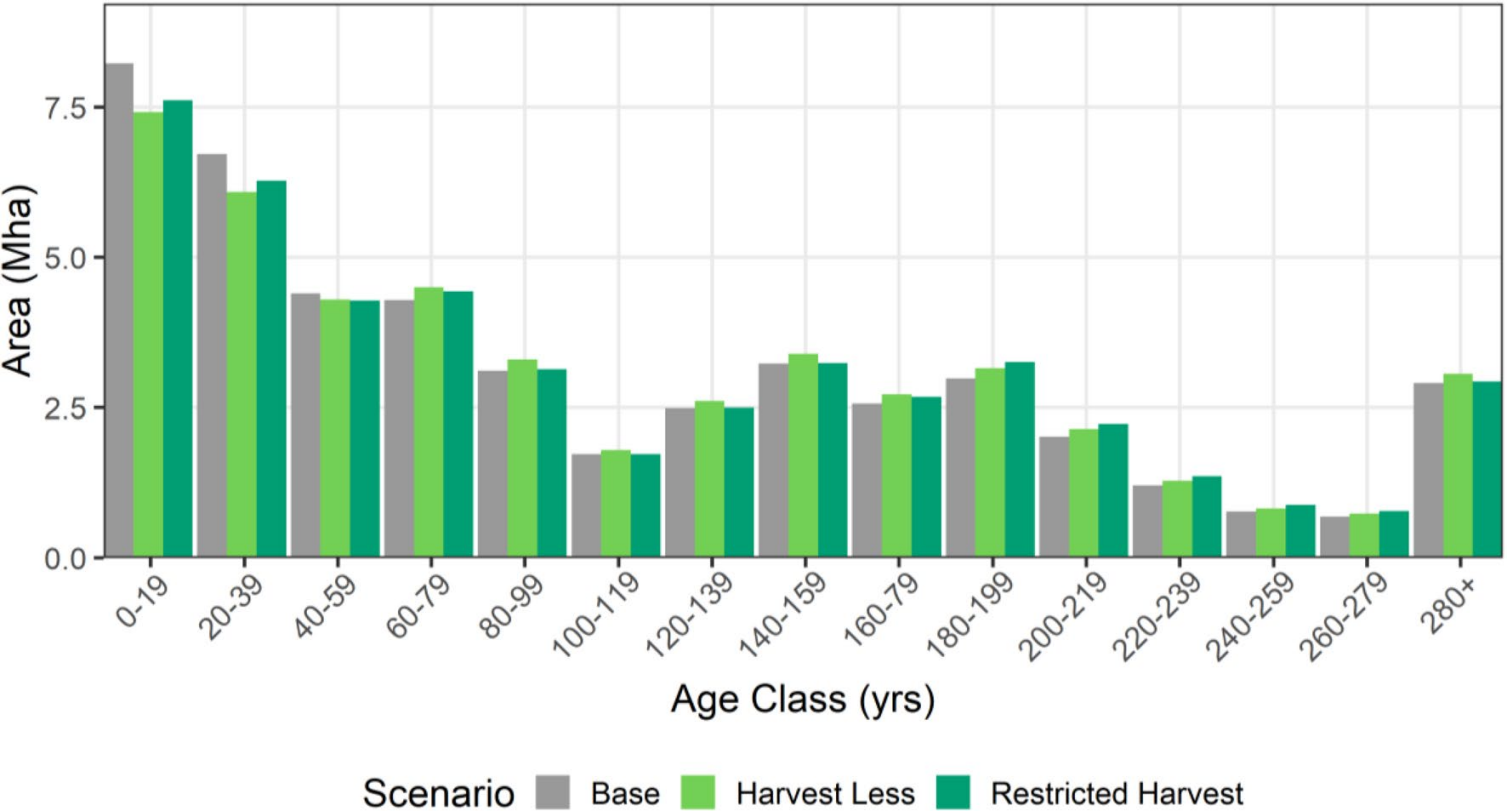
Age class distribution of stands within the timber harvesting landbase in 2070. Age classes for scenarios *Higher Recovery* and *Residues for Bioenergy* are the same as the *baseline* and are not shown

In addition to quantifying the impacts of mitigation activities on GHG reduction, we also estimated impacts on four other indicators: stand age, species, deadwood availability, and future timber supply. For forests eligible for harvest, the *Harvest Less* scenario had fewer stands less than 60 years old and more stands in all older age classes relative to the *baseline* (Fig. 6). The *Restricted Harvest* scenario also had fewer stands less than 60 years old, and more older stands, particularly within 180 to 240 years old.

Examining the species differences for young and old stands within forests eligible for harvest revealed the *baseline* scenario had a greater number of younger lodgepole pine and spruce stands and fewer older spruce, lodgepole pine and subalpine fir stands compared to the scenarios with lower harvest levels (Table 5).

**Table 5:** Species composition for the *baseline*, and differences by scenario for a) stands less than 60 years of age in 2070 and b) stands greater than 180 years within the timber harvesting landbase

Species	*Baseline* area in 2070 (Mha)	Change in area for *harvest less* minus *baseline* (Mha)	Change in area for *restricted harvest* minus *baseline* (Mha)
Stands less than 60 years of age			
Lodgepole pine	4.64	− 0.14	− 0.12
Spruce	2.23	− 0.10	− 0.13
Douglas-fir	1.29	− 0.07	− 0.04
Western hemlock	1.01	− 0.04	− 0.01
Subalpine fir	1.00	− 0.06	− 0.05
Aspen	0.74	− 0.04	− 0.04
White spruce	0.73	− 0.05	− 0.07
Engelmann spruce	0.54	− 0.03	− 0.02
Redcedar	0.40	− 0.02	0.00
Amabilis fir	0.20	0.00	0.00
Western larch	0.16	− 0.01	0.00
Other	0.41	− 0.02	− 0.02
Total	13.35	− 0.57	− 0.50
Stands greater than 180 years			
Spruce	0.94	0.07	0.12
Subalpine fir	0.77	0.04	0.04
Lodgepole pine	0.71	0.05	0.09
Douglas-fir	0.68	0.03	0.04
White spruce	0.51	0.03	0.07
Western hemlock	0.49	0.03	0.01
Engelmann spruce	0.33	0.02	0.02
Redcedar	0.29	0.02	0.00
Aspen	0.25	0.01	0.03
Black spruce	0.08	0.00	0.01
Amabilis fir	0.06	0.00	0.00
Other	0.17	0.01	0.01
Total	5.27	0.32	0.43

**Fig. 7:**
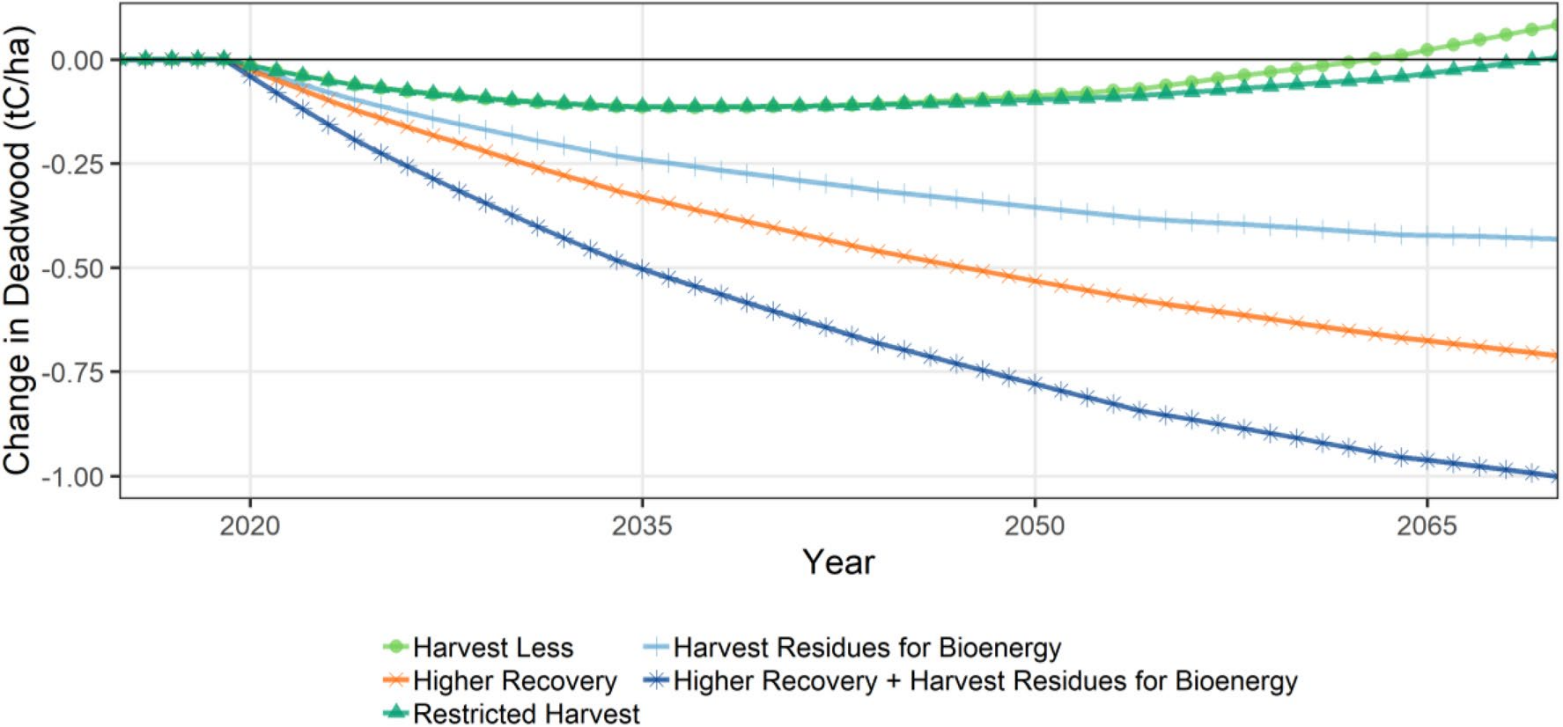
Change in deadwood density timeseries, relative to the *baseline*

For deadwood availability, the deadwood density had similar trends for the *baseline* and all mitigation scenarios (Additional file 1: Figure S7). Scenarios that used more of the harvested wood for products, or collected harvest residues for bioenergy had modest reductions in deadwood density relative to the *baseline* (Fig. 7) in the Timber Harvest Land Base (THLB).

**Fig. 8:**
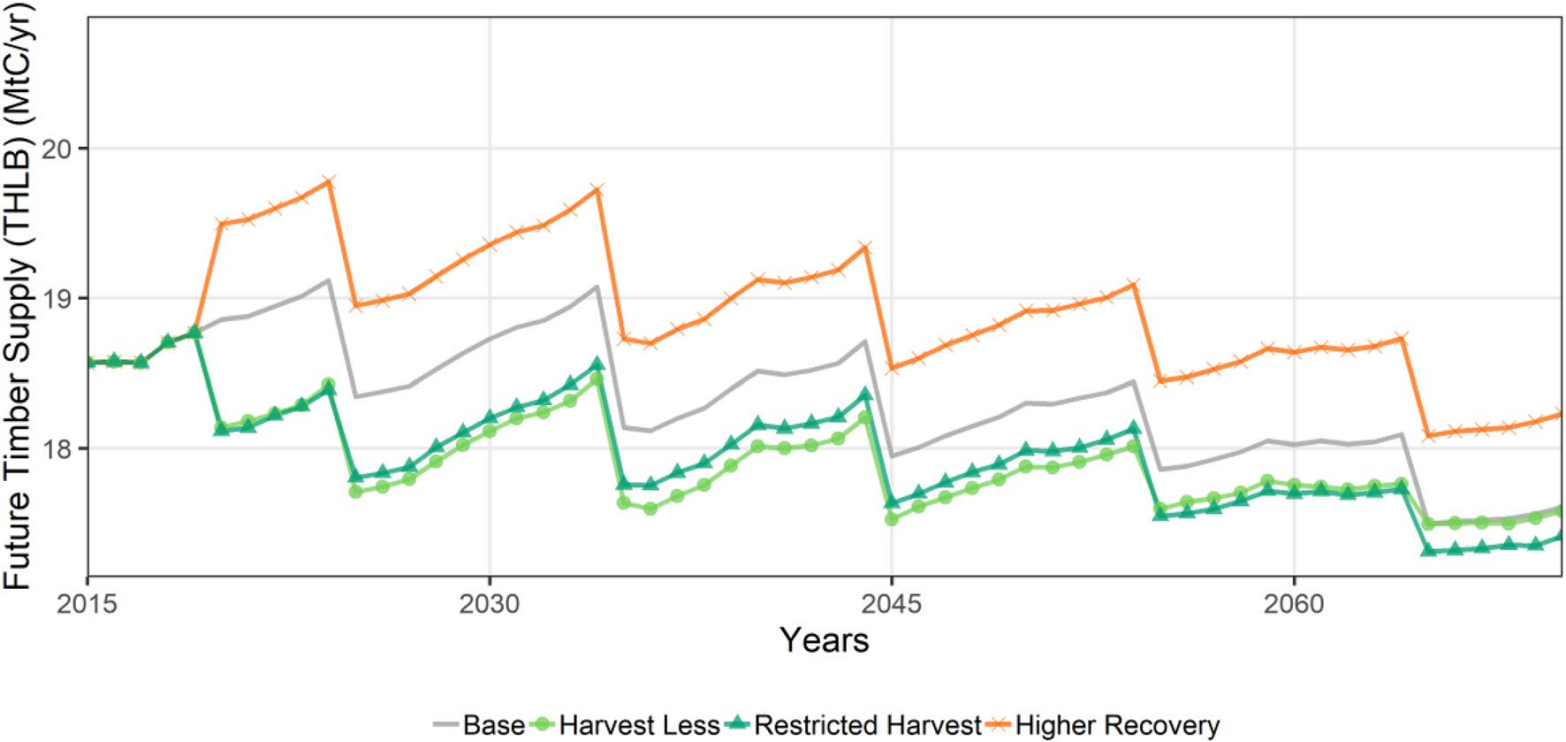
Timeseries of future timber supply within the timber harvesting landbase (THLB)

The fourth environmental indicator, future timber supply included harvested C combined with net merchantable growth within the THLB. The net merchantable growth component of future timber supply had decreasing net merchantable growth after 2050 (Additional file 1: Figure S8a), while the harvest transfers were fairly constant over time. Harvests had a decadal sawtooth pattern which reflected the decadal harvest schedule and the yield table interval (Additional file 1: Figure S8b). Future timber supply was highest for the *Higher Recovery* scenario followed by the *baseline* scenario, and then the two conservation scenarios (Fig. 8). The *Higher Recovery* scenario has the same forest growth expectation as in the *baseline*, but has higher future timber supply because more biomass is removed per unit of harvest area.

## Discussion

In our comparative analyses, every scenario we examined had secondary effects and uncertainties that are difficult to quantify. It is beyond the scope to bring in changes in growth and mortality associated with climate change, but some mitigation analyses have included these impacts for other countries [29, 34] and environmentally sensitive models are being developed [21] but are not yet implemented operationally. Biogeophysical contributions from changing harvest levels altering surface albedo were not considered, nor were biogenic volatile organic compounds, but these interactions may reduce the effectiveness of conservation scenarios [35].

Risk of reversal from wildfires was considered for conservation scenarios because severe fire seasons burned more than 2.5 Mha in British Columbia in 2017 and 2018 [54], and reserve status has been linked to wildfire probability in other regions [52]. Future severe fire seasons are expected for the interior and southern Cordillera of western Canada due to increasing temperatures [19, 63], high fuel loads from Mountain Pine Beetle after-effects [62], and reduced fuel moisture from changing weather patterns [64]. We estimated modest average reductions in the cumulative mitigation potential for conservation scenarios but acknowledge that burned area projections have a high uncertainty, and these results suggest that conservation-oriented scenarios in forests with low natural disturbance rates have the potential to reduce GHG emissions relative to harvesting, but will be of limited benefit in regions with high risk of natural disturbances. We did not assess the impacts of increased wildfire risk on the scenarios related to harvest utilization because we assumed the low projected harvest volumes (33 to 35 Mm^3^ year^−1^) would not be significantly affected by future wildfires, but this could be tested in future analyses. If wildfire and carbon models were more closely linked, the GHG impacts of fuel treatments (prescribed burning and salvage harvest) on future wildfire severity and burned areas could be investigated.

In addition to the uncertainty related to future wildfire risk, there is also uncertainty due to the use of merchantable yield tables to simulate forest growth. We used yield tables developed for unmanaged stands, which likely underestimates growth of stands planted after harvest for the *baseline* scenario. If managed stands achieve higher yields then the mitigation potential associated with conservation scenarios has been over-estimated. There is also uncertainty in growth of older stands for the conservation scenarios because yield tables based on even-aged stands simplifies their multi-story, multi-age, and multi-species characteristics. Old growth forests of the Pacific Northwest can be either C sinks or sources [12, 66]. In our analyses, conserved stands in the *Restricted Harvest* scenario were a small sink: net ecosystem productivity was roughly − 0.45 tC ha^−1^ year^−1^. Conserved stands in the *Harvest Less* scenario were a larger sink: − 0.69, − 0.84 and − 1.55 tC ha^−1^ year^−1^ for the northern interior, southern interior and coastal regions, respectively, because relatively more younger stands are conserved than in the *Restricted Harvest* scenario.

We considered low and high substitution benefits in the analyses because uncertainty in the substitution benefits contributes to uncertainty in mitigation results for energy [34] and products [50]. We found that provincial-level energy substitution benefits from future fuels were slightly smaller than those estimated from contemporary fuels (Fig. 4), but contemporary fuels had greater regional differentiation, specifically for regions with high industrial energy demand and low population, similar to the findings of an earlier study [23]. For future analyses, it would be beneficial to have spatial information on future community and industrial fuel consumption for each fossil fuel. In remote communities, fuel use is changing through several programs (the Clean Energy for Rural and Remote Communities (CERRC) program [39], the Indigenous Off-Diesel Initiative [40], and in 2018 the CleanBC plan [16] announced the goal to reduce by 2030 the diesel consumption in off-grid communities by 80%.

Uncertainty in the substitution benefits for wood products was assessed by using high and low substitution benefits for sawnwood and panels. A recent review of studies that have assessed substitution benefits for wood [33], found an average product displacement factor that is within the range of values used in this study, but additional information on displacement factors by commodity type and country would be useful, along with additional information on end-uses and associated product lifetimes (e.g. [5, 8]). Information on substitution benefits for pulp and paper is limited, and we assumed there was no substitution benefit, but given the proportion of C in this category (25% to 34% of wood commodities), refining these factors could have large impacts on the net GHG reduction. Regardless of the uncertainties about the actual magnitude of substitution benefits, our results clearly demonstrate that greater mitigation benefits can be achieved through policies that (1) increase the C retention time in harvested wood products by favouring long-lived over short-lived products including bioenergy, and (2) encourage the use of wood products to replace emission-intensive materials, e.g. in the building sector.

In terms of the economic analyses, similar studies have compared mitigation costs for various mitigation scenarios at the national scale [32] and for specific activities [45, 56, 68]. In this study, we used regionally differentiated economic assumptions by three broad regions (northern interior, southern interior, coastal region) as well as at the timber supply area (TSA) level for the *Bioenergy* scenarios in order to capture the spatial variation in market price and production cost (Additional file 1: Table S9). The cost and price assumptions associated with the bioenergy scenarios and the substitution effects were TSA-specific depending on residue availability, bioenergy facility type, transportation distance (simple estimates), and fuel mix. We assumed that log prices would be affected if harvest shifted among log grades due to mitigation scenarios. For example, the *Higher Recovery* scenario was assumed to increase the proportion of logs in lower grades and thus reduce overall average log prices, while the *Restricted Harvest* scenario was assumed to decrease the portion of top-grade logs, and therefore also reduce overall average log prices. However, no change in market prices of HWP was assumed in any scenario because HWP prices are usually determined by large-scale markets while log markets are relatively regional. Costs related to forest management were affected if harvest activities were altered by mitigation scenarios, for instance, logging costs increased in conservation scenarios because more dispersed cut blocks were needed to keep the same harvest characteristics (e.g., diameters, tree species, etc.). We also assumed a fixed $50/tCO_2_e carbon price over the entire period for slashburning as a penalty in the *baseline* to reflect a possible policy change to include slashburning in BC’s existing carbon pricing [17]. Manufacturing costs were also impacted by changes in production efficiency that then depend on the availability of input materials. Additional recovered fiber under *Higher Utilization* was assumed to be used in HWP following the same proportions as in the baseline, thus a lower manufacturing cost was assumed for pulp and paper production due to higher efficiency, but a higher manufacturing cost for solid wood products because of lower log quality. Similarly, higher manufacturing costs were assumed for all HWP in the conservation scenarios due to lower efficiency. In the *LLP* scenario, we assumed economy of scales increased manufacturing costs of pulp and paper (+ 2%) and decreased costs for solid wood products (− 2%) [67].

We found that the use of wood played an important role in determining the GHG reduction and cost per tonne in the *Higher Recovery* and *LLP* scenarios. Mitigation policies that seek to re-direct existing fiber flow to allocate additional fiber to wood products that have longer life span and can be used to substitute emission-intensive materials would be more cost-effective than using wood generally. We also found that, under our assumptions, greater fiber recovery per hectare would lead to higher mitigation potential and cheaper costs per tonne. Therefore, policy decisions that target the highest possible merchantable utilization rate would achieve the most cost-effective mitigation benefits, although limited mitigation potential was shown in the *Higher Recovery* scenario (Fig. 3). The cost per tonne in conservation scenarios showed little variation among implementation levels and displacement factors due to proportional changes between cumulative mitigation potential and total cost. Such an invariance occurred across most TSAs, indicating that the cost per tonne in conservation scenarios is relatively spatially independent. We found that net GHG reductions varied more by implementation level than the mitigation costs per tonne, particularly for the conservation scenarios.

The domestic portfolio, which was constructed by selecting scenarios that had the highest mitigation potential for each TSA, was found to be the most cost-effective scenario for the province. In general, the economic analysis suggested that about 85% and 70% of the total mitigation potential in the portfolio could be achieved below $50/tCO_2_e for the default and low implementation levels, respectively. This implies that, with appropriate actions, BC’s forest sector would be able to contribute significantly to climate change mitigation at costs that are below the carbon price of $50/tCO_2_e which will be implemented at the provincial level in 2021 and at the national level in 2022. The domestic portfolio only generated socio-economic benefits at the low implementation level, because at the default implementation level (Table 4), the portfolio consisted of more conservation scenarios, which negatively affect socio-economic benefits.

In addition to GHG impacts and costs, we also considered the impacts of mitigation activities on forest stand species, age-class distribution, deadwood and future timber supply. These factors are important for recreational, cultural, and economic values, as well as biodiversity indicators. In terms of species predominance by age classes, conservation scenarios in 2070 (default implementation level) had 8% more area in the 180+ aged stands than the *baseline* scenario that were mostly spruce, lodgepole pine and sub-alpine fir. In addition, conservation scenarios had fewer young stands (~ 4% fewer stands less than 60 years old) in 2070. These findings are consistent with studies from Sweden and Finland, where the area of old forest in the managed landscape increases if harvest levels drop [20, 42].

For changes in deadwood within the THLB (snags, downed logs, dead branches and dead coarse roots), overall changes were relatively small (< 1 tC/ha change relative to the C density in these pools of ~ 27 tC/ha in the *baseline* scenario). The mitigation scenario that collected harvest residues for bioenergy and products had the lowest levels of deadwood, consistent with previous analyses that found increasing harvest volumes decreased dead wood in managed forests compared with unmanaged forests [20]. Snags and coarse woody debris have been found to have high variability among and between the ecosystems related to natural disturbance types for both volume and decay class [57]. The amount of deadwood derived from pests and wildfires is significant in BC [31], and since the late 1990s mountain pine beetle (MPB) (*Dendroctonus ponderosae*) has killed over 700 Mm^3^ of merchantable timber and attacked a cumulative area of over 18 Mha [7].

Projected forest characteristics such as stand age, dominant species and deadwood availability could be used to further inform biodiversity indicators. It is beyond the scope of this analyses to assess the complex forest-wildlife interactions, but the spatially explicit model output at 1 hectare pixel resolution provides detailed information on the spatial extent of stand characteristics (age, species) and the availability of different types of dead wood (standing snags, coarse woody debris) which could be used to identify suitable habitats. For example, model output could inform habitats for woodpeckers which prefer large standing dead trees within dense canopies, or habitats for some predatory birds (owls, eagles, kestrals) that prefer trees in or adjacent to open areas, or large predator habitats that (e.g. cougar and wolverine) that use large cavities in coarse woody debris [28].

Mitigation scenarios that we did not consider in this analysis include afforestation, enhanced forest rehabilitation after natural disturbance, wildfire and forest management interactions, and adaptation scenarios that could have a mitigation benefit. Afforestation, which has been examined in previous studies [68, 69] and rehabilitation after natural disturbances can provide future C sequestration with other co-benefits such as greater long-term timber supply, and reduced fragmentation in wildlife habitats (e.g. [4]). Other scenarios that could be examined include cascaded wood use [13], salvage harvest in place of harvesting of live trees [49], and management of deadwood to reduce wildfire risk [6]. The secondary implications of any mitigation strategies for forest health, future fire risk and interactions with climate change impacts were not assessed here, nor were policy implications and public acceptance of mitigation actions, but these have been explored elsewhere [22, 44]. We assessed a limited number of scenario combinations, and additional scenario combinations at higher implementation levels could be analysed in future analyses using the existing quantitative framework that includes forest ecosystem, tracking of C in HWP, substitution benefits, economic and socio-economic indicators to identify GHG effective actions.

## Conclusions

We analyzed several mitigation scenarios and found that significant cost-effective mitigation by 2030, 2050 and 2070 with positive socioeconomic benefits would be possible if scenarios were implemented soon. Our analysis estimated that regionally differentiated portfolios provided the highest cumulative global and domestic mitigation by 2070 with combinations of activities related to the higher recovery of harvested merchantable biomass for products, the use of harvest residues for bioenergy in many regions, reduced harvest in low-disturbance regions, and greater use of longer lived wood products. This study is the first regionally differentiated mitigation study that considers biophysical, economic, and socio-economic impacts as well as other environmental indicators relating to forest species, age class, deadwood availability and future timber supply using a spatially explicit framework applied at 1 hectare resolution to all of BC’s public forests. The analyses conducted in this study contribute to the global understanding of forest sector mitigation options by providing an integrated framework to synthesize the methods, assumptions, datasets and models needed to quantify mitigation activities using a systems approach. An understanding of economically feasible and socio-economically attractive mitigation scenarios along with trade offs for environmental indicators relating to species composition and age, helps decision makers with long-term planning for land sector contributions to GHG emission reduction efforts, and provides valuable information for stakeholder consultations. Challenges remain, however, in the quantification of climate change impacts, including changes in future tree growth and mortality rates and changes in future wildfire risks.

## Methods

Our analysis assessed the net GHG reduction resulting from changes in forest management, the use of wood products or bioenergy, and substitution benefits achieved through wood product uses. We defined forest sector climate change mitigation based on C stock changes in the forest ecosystem and emissions associated with the use and disposal of products manufactured from wood that was harvested within the BC, regardless of where in the world these products would be consumed—in accordance with the general framework of the Production Approach, as described in the 2006 IPCC Guidelines [24] which Canada has implemented for international reporting [11]. We did not consider leakage effects due to imported wood products, which we assumed were minimal at the provincial scale because softwood lumber imports to BC are < 0.05% of lumber exports from 2013 to 2016 based on international trade flows [55]. Domestic mitigation was defined as the forest sector mitigation plus substitution benefits resulting from the use of HWP in BC, and global mitigation was defined as domestic mitigation plus substitution benefits from elsewhere wood harvested in BC was used, including within Canada, but outside of BC.

### Ecosystem C modeling

Forest ecosystem C dynamics were estimated using the Generic Carbon Budget Model (GCBM), a C budget model that uses the same pools structure and is based on the equations, logic and default assumptions of the well-established Carbon Budget Model of the Canadian Forest Sector (CBM-CFS3) [30]. The GCBM is built on the open-source platform of the Full Lands Integration Tool (FLINT) developed and maintained by moja global (http://moja.global). The GCBM is a spatially explicit modeling environment where data input and model parameters are based on spatial layers combined with aspatial information such as yield tables, Additional file 1: Figure S1. The model was run from 1990 to 2070 on all public forests within BC at 1 ha (0.001 degree) resolution.

**Fig. 9:**
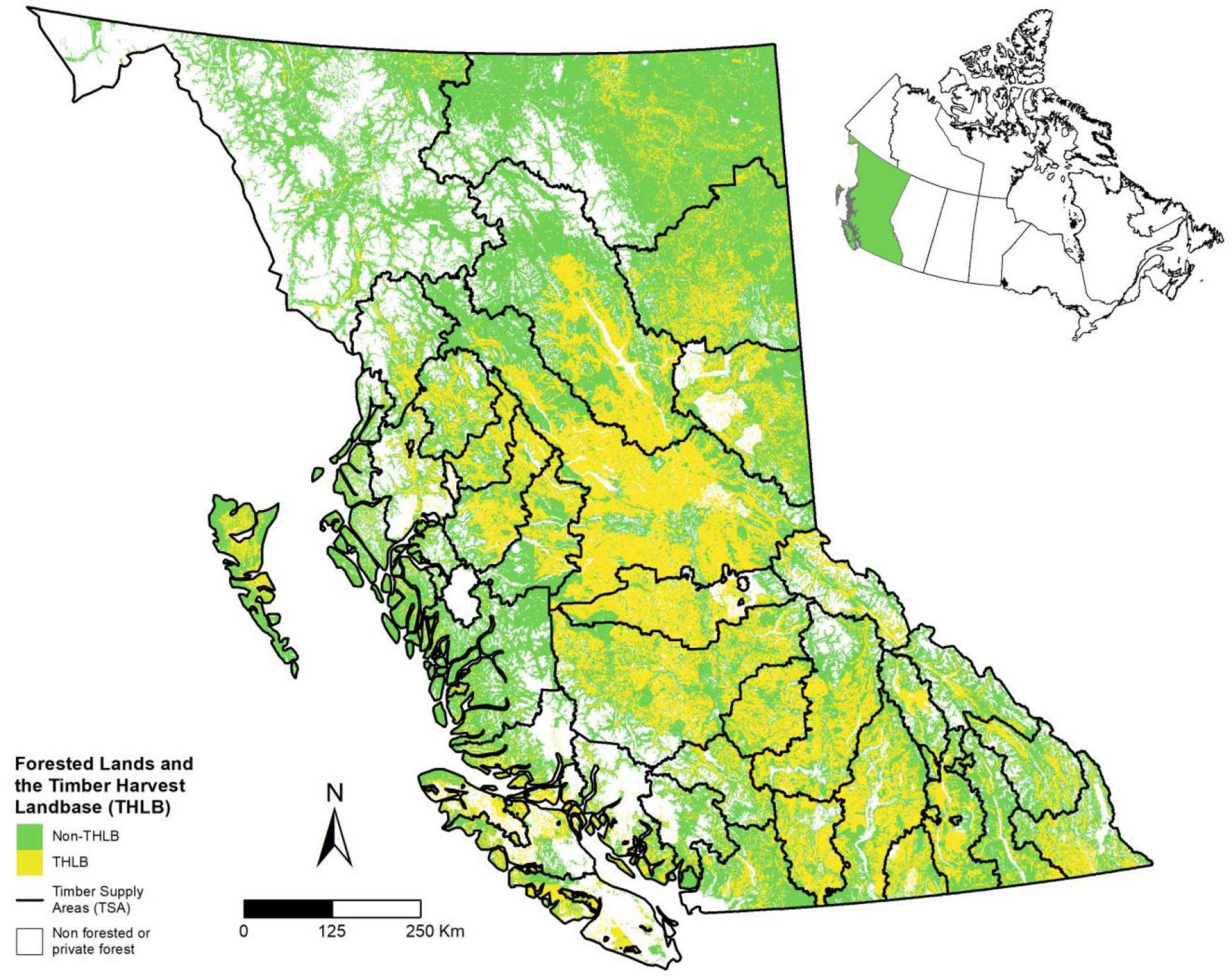
Map of forested land including the timber harvest landbase designation (THLB) and Timber Supply Area (TSA) boundaries. Mitigation scenarios were applied to forest management activities within the timber harvesting landbase, and the entire forested landbase was simulated. Inset map of Canada identifies the province of British Columbia (BC)

Forest inventory and yield table datasets for BC’s public forests were provided by the BC Ministry of Forests, Lands, Natural Resource Operations and Rural Development (FLNRO) (Additional file 1: Table S1). The 2015 spatial forest inventory included information on leading species, age, site index, and harvest eligibility. Of the modeled 62.9 Mha of public forest, 22.6 Mha was within the Timber Harvest Land Base (THLB) and eligible for harvest, Fig. 9. Leading species within the THLB were mostly coniferous species: lodgepole pine (29.7%), spruce (16.0%), Douglas-fir (11.8%), subalpine fir (8.9%), western hemlock (7.3%) and aspen (7.1%).

Forest disturbances from 1990 to 2014 were modeled using clearcut harvest cutblocks and natural disturbances (wildfire and mountain pine beetle (*Dendroctonus ponderosae* Hopkins) (Additional file 1: Table S1). Future wildfires (from 2015 to 2070) were assumed to be 77.6 kha year^−1^ annually for the province, estimated from the average of the historical burned area for each Timber Supply Area (TSA) from 1990 to 2014 and applied at the TSA level. Future harvest levels (from 2015 to 2070) were estimated from Annual Allowable Cut levels [15] for public lands, reduced by 15% because harvest levels are often lower than allowable levels, and further reduced in three regions with large impacts of 2017 fires. Large fires also occurred during 2018, but these analyses were started before the 2018 wildfire season. Scheduling of spatial harvest and wildfire from 2015 to 2070 was accomplished using a harvest scheduler with random fire.^1^ Clearcut harvesting assumed utilization rates of 85% of the merchantable stem biomass present at the time of harvest, with the remainder left on site as logging residue, along with trees below merchantable size. A portion of harvest residues in the *baseline* were piled and burned for fuel hazard management. For four regions, harvest utilization rates where reduced from the standard 85% level: Timber Supply Area (TSA) 4—Cassiar 78%, TSA 10—Kalum 74%, TSA 38—Arrowsmith 70%, TSA 43—Nass 27%; based on the 2015 billing information for unavoidable harvest waste (2018, *personal communication,* FLNRO).

### Harvested wood products modeling

Carbon transferred from forest ecosystems to products and bioenergy was tracked through manufacturing, export, use, and post-consumer treatment by the Carbon Budget Modeling Framework for Harvested Wood Products (CBM-FHWP) [11, 49] (see Additional file 1). Export rates of roundwood were based on information in the 2014 BC Mill report [14]: 27.6% for the coast and 1.3% for the interior. Default lifetimes were assumed for HWP commodities: sawnwood and other industrial roundwood had a 35 year half-life, panels had a 25 year half-life, and pulp and paper had a 2 year half-life [25]. Post-consumer commodities were sent to landfills, or incinerated, or used for energy (Additional file 1: Table S1). A portion (0.6) of solid wood and paper products that were sent to domestic landfills was assumed degradable with half carbon dioxide and half methane emissions (Additional file 1: Table S1), and some of the methane was captured and flared and/or used for energy [11].

### Substitution impacts

Two substitution impacts were included: substitution between solid wood products and emissions-intensive materials, and substitution between bioenergy and fossil fuels used in stationary combustion to produce power, combined heat and power, or heat. Substitution benefits for solid wood products considered emissions associated with extraction, transportation of raw materials, and manufacturing, and were previously estimated for Canada assuming a series of end-use products (e.g. single-family homes, furniture, etc.) weighted by consumption, and emissions from alternative non-wood end-use products [48]. Two levels (high and low) of substitution benefits were applied to assess the impact on the net change in GHG emissions. Low substitution benefits assumed a broad range of end-use products and a range of non-wood alternatives, hereafter referred to as ‘General Use’, where we assumed 0.54 tC emissions were avoided per tC of sawnwood used, and 0.45 tC emissions were avoided per tC of panels used [48]. High substitution benefits assumed incremental wood products were used only for building construction and were assumed to substitute for steel and concrete, hereafter referred to as ‘Wood Buildings’, where avoided emissions were 2.1 tC and 2.2 tC for 1 tC of sawnwood and 1tC of panels, respectively [67].

Substitution benefits from bioenergy were estimated using a linear programming (LP) model, which maximized avoided emissions from using harvest residues for electricity and heat production by selecting from nine different candidate bioenergy facilities (Additional file 1: Table S13) to substitute for the highest emissions baseline fuels [48]. We used two *baseline* fuel assumptions, as described in Additional file 1. High substitution benefits, hereafter referred to as ‘Contemporary Fuels’, were based on spatially explicit contemporary fuels from communities [2], remote communities [46], and industry [1], Additional file 1: Tables S3 and S4. Low substitution benefits, hereafter referred to as ‘Future Fuels’, were based on a low-C electricity forecast that assumed higher carbon prices and greater adoption of emerging energy technologies [41]. We assumed that new bioenergy facilities would be constructed, but did not include emissions associated with facility construction because we assumed fossil energy sources would have similar construction or renovation emissions.

### Mitigation scenarios

Five forest management scenarios were assessed at three activity implementation levels, relative to the *baseline* (Table 6). Two conservation scenarios were considered. The first scenario, *Harvest Less*, reduced the harvest area by ten percentage points, while the second scenario *Restricted Harvest*, reduced harvest levels of older stands, where the age threshold was defined by the natural disturbance regime for each biogeoclimatic ecological subzone (Additional file 1: Table S5). The third scenario, *Higher Recovery*, kept the harvest area unchanged, but increased the recovery rate of harvested merchantable stemwood by five percentage points, which increased the harvest volume per hectare, and reduced the amount of harvest residues and their related decay and/or slashburning emissions. The incremental harvest volume was assumed to be used for the same product mix as the original harvest. In the fourth scenario, *Harvest Residues for Bioenergy*, harvest levels and recovery (utilization) rates were the same as the *baseline* scenario, but slashburning was stopped, and 25% of harvest residues (including branches, small trees, unused merchantable-sized trees and snags) was collected and transported to hypothetical bioenergy facilities to produce heat and/or electricity in place of using fossil fuels. Four TSAs with lower harvest utilization rates were not included in the *Harvest Residues for Bioenergy* or conservation scenarios because these scenarios were implemented assuming standard harvest utilization rates. Two scenarios that involved harvest residue management were combined into a fifth scenario (*Higher Recovery plus Harvest Residues for Bioenergy*) which first increased the use of C from merchantable-sized trees for products, and then used a proportion of remaining residues for bioenergy production. The only wood-use scenario, a *Longer*-*Lived Products* (*LLP*) scenario, shifted by six percentage points the wood fibre used for pulp and paper in the *baseline* to panels and sawnwood. The shift in commodities extended the retention period of C in HWPs and accrued substitution benefits from the incremental production of sawnwood and panels. The *LLP* scenario was also combined with each of the forest management scenarios to determine the combined mitigation benefits.

**Table 6:** Scenario parameters for the *baseline* and mitigation scenarios (default implementation level with low and high implementation levels given in parenthesis

Parameter	Unit	*Baseline*	*Higher recovery*	*Harvest less*	*Harvest residues for bioenergy*	*Higher recovery and harvest residues for bioenergy*	*Restricted harvest*
Forest ecosystem							
Harvest recovery	Utilization of c in stemwood from merchantable-sized trees (%)	85^a^	*90 (88,93)*	85	85	*90 (88,93)*	85
Harvest residue mgmt	Piled and burned (percent of area)	50^b^	50	50	*0*	*0*	50
Harvest residue mgmt	Collected for bioenergy (percent of residues)	0	0	0	*25 (20,30)*	*25(20,30)*	0
Area excluded from harvest	Harvest area (percent change)			*−10 (− 2, − 20)*			Based on age threshold

Parameter	Unit	*Baseline*^*c*^	*Longer*-*Lived Products*^*d*^
HWP			
Sawnwood production	% of total products	51.6	*54.6 (53.1, 56.1)*
Panels production	% of total products	18.9	*21.9 (20.4, 23.4)*
Other industrial RW production	% of total products	2.5	2.5
Pulp and paper prod.	% of total products	27.1	*21.1 (24.1, 18.1)*

Commodity	General use (tC/tC)	Wood Buildings (tC/tC)
Product substitution benefits		
Sawnwood	0.45	2.2
Panels	0.54	2.1
Other solid wood	0	0
Pulp and paper	0	0

Text in italic indicates a change from the *baseline*

^a^Utilization rates for 4 TSAs were assumed to be lower than 85%

^b^Slashburning percentage for coast regions were 15%

^c^Proportions for 2030 + are listed. In 2016, commodity percentages were 34.3% pulp and paper, 47.1% sawnwood, 16.1% panels, and 2.5% other industrial roundwood production. Baseline percentages were assumed to decrease Pulp and Paper from 2016 until 2030, with corresponding increases in sawnwood and panels

^d^Longer-Lived Products (LLP) commodity proportions were implemented starting in 2020, and assumed constant proportions until 2070

### Environmental ecosystem indicators

We examined the change in four additional environmental indicators for forests within the THLB: area of forest tree species, deadwood density, forest age-class distribution, and future timber supply for each of mitigation scenarios, relative to the *baseline*. The area of forest species was estimated for young stands (ages less than 60 years) and mature stands (ages greater than 180 years). Deadwood density (defined as the tC per unit ha) was estimated as the sum of C in standing dead trees (snags) and associated branches, coarse woody debris (CBM-CFS3’s medium pool), and dead coarse roots within the mineral soil. Future timber supply was estimated as the sum of future harvest C and the net merchantable increment C (gross merchantable growth minus annual merchantable mortality).

### Risk of reversal for conserved stands

Ecosystem modeling of conserved stands, i.e. those that did not get harvested as a consequence of mitigation action, assumed that there was no risk of reversal from pests, wildfires or drought, which overestimates the ecosystem sequestration potential [60]. We assessed the risk of reversal *ex*-*post* by overlaying maps of conserved stands with 100 Monte Carlo draws of spatially explicit future fires (based on methods by Metsaranta et al. [36]). Stand-replacing high severity fire maps were based on fitted log-normal distributions to historical data from 1950 to 2018, that were randomly placed on the forested landscape, and with an assumption that the annual area burned would double over 50 years (see Additional file 1). The percent of conserved stands that would burn was estimated annually for each of the 100 draws based on the area of conserved stands that burned divided by the cumulative conserved stand area. The average percentage (from 100 draws) of affected stands was applied as a reduction factor ex-post to the annual forest mitigation potential from the burn year until 2070. This assessment does not take into consideration secondary effects, such as changes in landscape-level fire risks associated with a larger proportion of older stands.

### Portfolios and normalized mitigation estimates

Portfolios were constructed for each of the three implementation levels by selecting the scenario with the greatest reduction in net GHG emissions for each region and then summing all regions. A domestic portfolio was estimated from the cumulative mitigation within BC, and a global portfolio included the domestic and foreign mitigation potential. The available selection of scenario and scenario combinations included *Harvest Less*, *Higher Recovery*, *Harvest Residues for Bioenergy*, *Higher Recovery *+* Harvest Residues for Bioenergy*, *Restricted Harvest*, and all scenarios and the *baseline* with *LLP.* Scenarios implementation levels were selected independently of each other, which can affect the selection of scenarios included in the portfolio. To avoid bias introduced by the independent implementation levels, we examined normalizing the net change in GHG emissions based on an earlier study that found normalizing by forest area or mitigation activity area facilitated scenario comparisons [47]. We assumed the change in recovered harvest biomass, which included changes in harvest levels and harvest residues for bioenergy, would be a suitable normalization factor. The normalized mitigation potential was estimated by linearly regressing log base ten of the cumulative net GHG reduction for each region in 2070 by log base ten of the magnitude of the cumulative change in harvest biomass transferred to the forest product sector, relative to the *baseline*, for each mitigation scenario.

### Mitigation costs and socio-economic indicators

Mitigation costs were estimated using the Model for Economic Analysis of Forest Carbon Management (MEA-FCM) which has been used at both the national [32] and provincial level [67]. Mitigation cost was defined as the change in the present value of the net revenue (NR) of both the forest sector (FS) and interacting product industry and energy sectors affected by substitution (SUB),

1 $$Cost = \Delta NR_{FS} + \Delta NR_{SUB}$$

Net revenue of the forest sector was defined as the total revenue minus the total costs for forest management activities including harvesting, residue management, wood product manufacturing and bioenergy production. The change in net revenue in the forest sector was calculated by taking the difference between the *baseline* and mitigation scenario. The change in net revenue in interacting product and energy sectors affected by substitution was defined as

2 $$\Delta NR_{SUB} = \mathop \sum \limits_{j = 1}^{3} \left( {p_{j} - c_{j} } \right)u_{j} \Delta HWP_{j}$$

where subscript *j* refers to the three products substituted by wood (concrete and plastic that were substituted by sawnwood and panels, and fossil fuel energy substituted by bioenergy from harvest residues), *p* and *c* refer to the per unit prices and costs, respectively, *u*_*j*_ represents the amounts of alternative products or fossil fuel energy that were substituted by one unit of wood products or harvest residues, and ∆*HWP* is the quantity change in wood products or harvest residues for the mitigation scenario relative to the *baseline*. The cost per tonne was then calculated for each scenario by dividing the cumulative mitigation cost in each region by the cumulative mitigation potential, assuming a 3% discount rate for mitigation costs [58] and a 1% discount rate for the mitigation potential [67]. Prices and costs were developed in consultation with FLNRORD and FPInnovations and are given in Additional file 1: Tables S9–S15. Historic log prices of 5-year average (a business cycle) and annual average prices for HWP after the economic recession in 2009 were used in the analysis to reflect the normal long-term price levels. Recent historic logging costs (to reflect recent practices) and post-2009 manufacturing costs were employed. A $50/tCO_2_e penalty for slashburing has been assumed in the *baseline*, in addition to the $5/odt burning cost. We did not estimate mitigation costs and socio-economic impacts for the high implementation level of the *Harvest Less* scenario, because a 20% harvest area reduction would result in fundamental changes in the industrial structure and mill closures, and would require a different set of economic assumptions.

The socio-economic impacts of mitigation scenarios on employment, GDP, and government revenues in BC’s economy were estimated from multipliers from Canada’s input–output (I/O) model [53], as described by Xu et al. [67]. Multipliers and labor intensity assumptions used for job estimates are given in Additional file 1: Tables S16 and S17.

## Supplementary information

**Additional file 1:** Supplementary information on biophysical and economic modeling

**Additional file 2:** Supplementary information on bioenergy optimization modeling

## Data Availability

The forest inventory dataset supporting the conclusions of this article is available in the Province of British Columbia’s Forest Inventory repository https://www2.gov.bc.ca/gov/content/industry/forestry/managing-our-forest-resources/forest-inventory.

## Abbreviations

BC: British Coumbia
C: Carbon
CBM-CFS3: Carbon budget model of the Canadian forest sector v3
GCBM: Generic carbon budget model
GDP: Gross domestic product
GHG: Greenhouse gas
FTE: Full time equivalent
HWP: Harvested wood products
LLP: Longer lived products
SB: Substitution benefits
THLB: Timber harvest land base
TSA: Timber supply area

## Footnotes

1 Paradis 2018. DEG: A disturbance event generator for GCBM, Internal report.
