# Supplementary material for: Climate change mitigation in British Columbia’s forest sector: GHG reductions, costs, and environmental impacts
Source: Carbon Balance Manag. 2020 Oct 1;15:21. doi: 10.1186/s13021-020-00155-2 (PMC7531139; doi:10.1186/s13021-020-00155-2)
Supplement: Supplementary file 1 — Additional file 1: Supplementary information on biophysical and economic modeling [file 13021_2020_155_MOESM1_ESM.docx]

Additional file 1 for:

Forest sector climate change mitigation in British Columbia: GHG reductions, costs, and environmental impacts

C. Smyth, Z. Xu, T. C. Lemprière, and W. A. Kurz

Contents

[Ecosystem and Harvested Wood Products C Modeling 1](#_Toc34888167)

[*Baseline* fossil fuel demand 6](#_Toc34888168)

[*Restricted Harvest* Age Thresholds 8](#_Toc34888169)

[Mitigation scenarios 13](#_Toc34888170)

[Future Fire Risk: Monte Carlo Fires 18](#_Toc34888171)

[Economic Prices and Costs 22](#_Toc34888172)

[Socio-economic Multipliers 30](#_Toc34888173)

[Mitigation Cost Estimates and Socio-economic Impacts 31](#_Toc34888174)

# Ecosystem and Harvested Wood Products C Modeling

The workflow for estimating spatially explicit forest ecosystem GHG emissions is shown in Figure S1. Pre-processing workflow steps included assembling spatial layers, projecting them to WGS84 format at 0.001 degree resolution, and combining the spatial information with aspatial information such as yield tables and transition rules. Post-processing steps combined raster output from the GCBM into a time series stack for each C indicator specified as output.


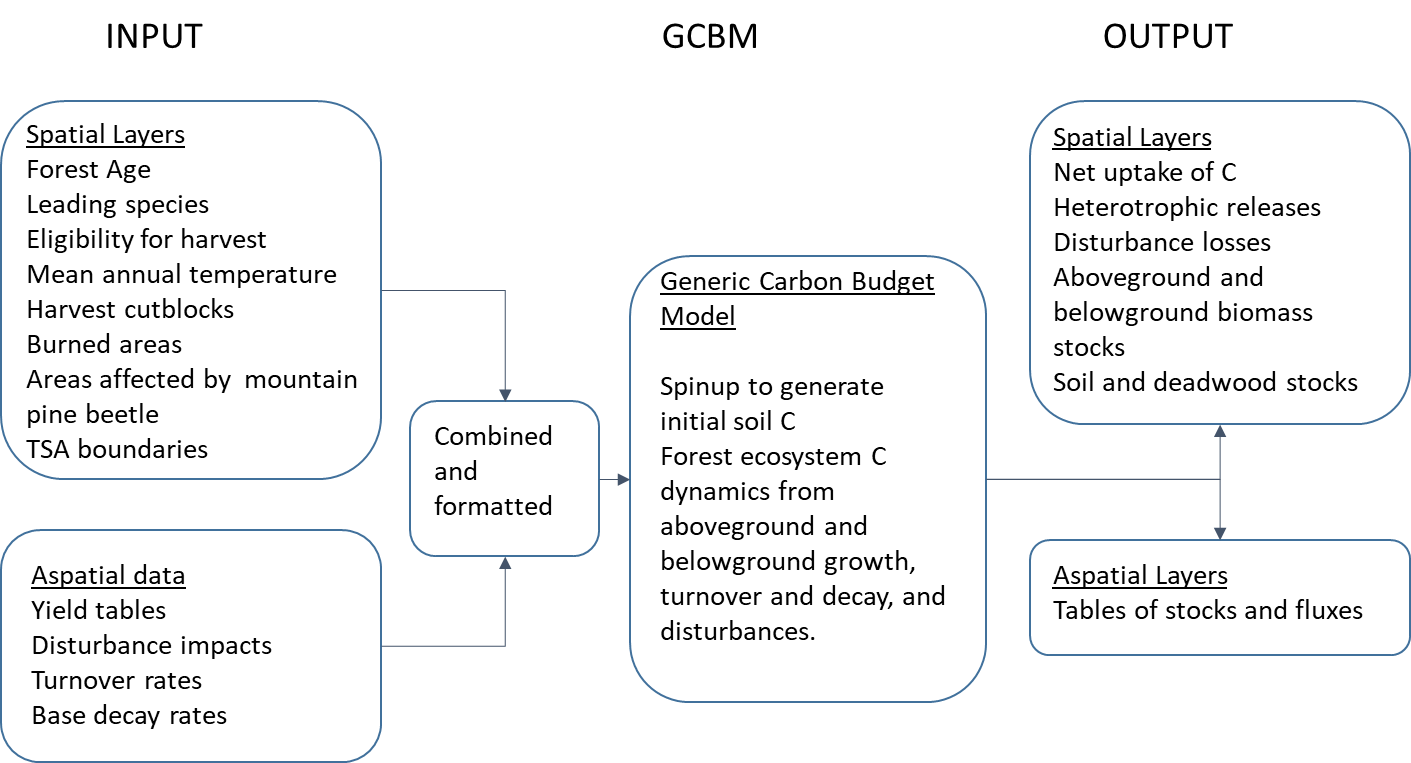


*Figure S1. Generic Carbon Budget Model workflow for spatially explicit carbon budget modelling.*

Inventory and baseline parameters for GCBM and Harvested Wood Products (HWP) modeling is shown in Table S1.

The 2015 Vegetation Resource Inventory (VRI) dataset supplied by BC Ministry of Forests, Lands, Natural Resource Operations and Rural Development (BC FLNRO) was used for the GCBM modeling (<https://catalogue.data.gov.bc.ca/dataset/vri-forest-vegetation-composite-polygons-and-rank-1-layer>). Stand-specific growth curves were supplied that were associated with the species, site class and biogeoclimatic ecosystem classification of the VRI data. Spatial datasets for forest disturbance were also provided for harvest (clearcut), wildfires and mountain pine beetle. Wildfire and mountain pine beetle datasets were consistent with natural disturbance layers used in international reporting tools for Canada’s greenhouse emissions from the forest sector (<http://unfccc.int/national_reports/annex_i_ghg_inventories/national_inventories_submissions/items/10116.php>).

*Table S1. Study region characteristics, forest inventory information and baseline assumptions.*

| Category | Description | Parameter Value |
| --- | --- | --- |
| Forest Inventory | Total area  Management Units  Inventory Vintage  # records (~100m^2^, 1 ha pixels)  Merchantable yield tables | 62.9 Mha  Timber Supply Area (TSA)  2015  67.5 k  Gross merchantable volume (VDYP7) based on species, site index, and BEC zone |
| Projected Activity Data | Harvest amount  Harvest methods  Wildfire average 1990 to 2014  Land use change | 33 to 35 Mm^3^ yr^-1^  85% utilization rate^a^, slashburn 50% of harvested area in the interior, 15% on coast  77.6 kha/yr  None |
| Harvested Wood Products | Bioenergy from roundwood  Roundwood export  Milling efficiency  Mill residues  Commodities (2030+)  Sawnwood  Panels  Pulp and paper  Other industrial roundwood  Commodity export rates  Sawnwood exported  Panels exported  Pulp and paper exported  Other industrial roundwood  Retired products  Sawnwood  Panels  Pulp and Paper  Other industrial roundwood  Landfill decomposable fraction  Landfill decay half-life  Landfill decomposition emissions gases Landfill CH4 flared or energy production | 0%  1.3% (Interior) 27.6% (Coast) of roundwood  71.9% of remaining roundwood used for commodities  28.1% industrial bioenergy  51.6% of total commodities  18.9%  27.1%  2.5%  62.6% of all sawnwood exported  53.9%  93.9%  0%  20.3% incinerated, remainder to landfill  11.9% incinerated, remainder to landfill  24.5% incinerated, remainder to landfill  100% incinerated  0.6  8.4 years  50 % CO_2_ 50 % CH4  Up to 34% of CH4 produced |
| Energy displacement factor | Bioenergy from mill residues | 0.5 tCO_2_e/tCO_2_e |

^a^ Four regions had lower harvest utilization rates: Cassiar 78%, Kalum 74%, Arrowsmith 70%, Nass 27%.

A spatial rollback tool adjusted the forest inventory from 2015 to a baseline year of 1990. For stands older than 26 years, the forest age was adjusted downwards. For younger stands, a wildfire or harvest disturbance event was applied at the stand establishment year, and the pre-disturbance stand age was randomly chosen from age distributions constructed for harvest and fire disturbances (Table S2). A regeneration delay was applied if there was a temporal gap between the inventory establishment year and the disturbance year. If a corresponding disturbance was not found, then the stand was assumed to be harvested in the establishment year.

*Table S2 Assigned pre-disturbance age distributions for stands that were disturbed within the 1990 to 2014 period for a) wildfire and b) harvest.*

| 1. Wildfire |  | 1. Harvest |  |
| --- | --- | --- | --- |
| Age | Proportion | Age | Proportion |
| 30 | 0.1 |  |  |
| 40 | 0.1 |  |  |
| 50 | 0.1 | 50 | 0.35 |
| 60 | 0.1 | 60 | 0.3 |
| 70 | 0.1 | 70 | 0.25 |
| 80 | 0.1 | 80 | 0.05 |
| 90 | 0.1 | 90 | 0.03 |
| 100 | 0.1 | 100 | 0.02 |
| 110 | 0.05 |  |  |
| 120 | 0.05 |  |  |
| 130 | 0.05 |  |  |
| 140 | 0.05 |  |  |

Scheduling of spatial harvest and wildfire from 2015 to 2070 was accomplished using a Disturbance Events Generator (Paradis, 2018). An adjustment was made to the inventory to take into account the 2017 wildfires which burned 1.2 Mha. Inventory records affected by severe or very severe wildfires (based on burn maps provided by Ann Morrison, FLNRO) were removed from the harvest eligibility list.

# *Baseline* fossil fuel demand

In this analysis, we used contemporary spatially explicit community and industrial energy use by fuel type (BC MOECCS 2015, 2016), in conjunction with gap-filled remote community fuel use (RECD, 2017) to define the *baseline* fossil fuel assumptions for the high substitution benefits case. The low substitution benefits case assumed future fuels scenario for the *baseline*, for which the electricity fuel mixes was calculated assuming a future provincial scenario with higher carbon prices and greater adoption of emerging energy technologies (NEB 2017).

*Contemporary Fuels*

Community energy use in the Community Energy and Emissions Inventory included direct fuel emissions from residential, commercial, and small or medium industrial users across BC. Utility providers contributed information on electricity, natural gas and piped propane, with additional information provided for heating oil, delivered propane and wood burning. Emissions from large industrial users ( >10 ktCO_2_e yr^-1^), associated with stationary combustion from BC’s industrial facility GHG emissions data, were included in the *baseline* where bioenergy could be a suitable substitute (which excluded waste treatment facilities and landfills. For this analysis, we assumed that bioenergy would not be used for fossil fuels burned in industrial greenhouses and forestry mills (saw mills, pulp or paper mills). Industrial fuel types were assigned based on the ratios of methane to nitrous oxide emissions, according to Table S3. Once the fuel types were assigned (fuel oil, propane, diesel, coal, natural gas) we assumed one third of industrial energy demand was used for electricity, and two thirds for heat (Howard and Smyth 2018; Smyth et al. 2017a).

*Table S3. Fuel types assigned to methane to nitrous oxide ratio ranges.*

| Fuel type | CH4:N2O range |
| --- | --- |
| Light fuel oil | <0.208081 |
| Propane | >0.208081 and <0.291361 |
| Diesel | >0.291361 and <0.431936 |
| Residual fuel oil | >0.431936 and < 0.609354 |
| Coal – electric utilities | > 0.609354 and < 0.712157 |
| Light fuel oil | > 0.712157 and < 0.947705 |
| Coke oven gas | > 0.947705 and < 1.057456 |
| Natural gas | > 1.057456 and < 1.310976 |
| Coal – industrial heat | > 1.310976 and < 1.687583 |
| Residual fuel oil - industrial | > 1.687583 and < 3.200695 |
| Petroleum coke | > 3.200695 and < 5.502301 |
| Fuel oil – electric utilities | > 5.502301 and < 7.903004 |
| Natural gas | > 7.903004 and < 154.1507 |
| Coal - residential | > 154.1507 and < 200 |

All communities, industries, and remote communities had geo-referenced coordinates that were overlaid with TSA boundaries to estimate the regional energy use. Linear facility larger emitters (pipelines) had no point location and were assumed to be co-located with its associated individual facility location.

*Future Fuels*

For the *Future Fuels* scenario, energy fuels were determined from an NEB electricity forecast assuming higher carbon prices and greater adoption of emerging energy technologies, heating fuels and remote community information. The energy demand was estimated from the population within a TSA multiplied by a per capita emissions estimated from 2016 (NEB 2016 electricity generation for BC divided by the 2016 census population). Fuel mixes from NEB were estimated for electricity fuels as an average of the fuel mixes from 2005 to 2040, and heating fuels, where projections were unavailable, used the 2014 heating fuels. The provincial NEB electricity fuel mix was then combined with remote communities information to determine a regional fuel mix. Missing population information in the remote community database was estimated by overlaying the 2016 census information, with a maximum population threshold of 100. The TSA-level electricity fuel mix was estimated by multiplying the share of the population connected to a grid by the NEB electricity mix and adding the share of the population within the remote community population multiplied by the fuel mix.

Summary statistics for the two *baseline* fuel assumptions are given in Table S4, and TSA-level information is provided in the Supplementary Bioenergy Material. Contemporary heating fuels had higher variability than future fuels because community and industrial fuels use varied spatially across the province, consistent with findings from an earlier analysis (Howard and Smyth 2018). Electricity emissions were generally lower in the future fuels scenario than in the contemporary fuels scenario, because low-emissions grid electricity was assumed to be used by industry and communities (except remote communities).

*Table S4. Baseline heating shares by fuel type for TSAs and emissions intensities.*

|  | Heating fuel | Percent of TSA(%) | Emissions Intensity  (kgCO_2_e/MWh) |
| --- | --- | --- | --- |
|  |  | Median (25%., 75%.) |  |
| *Contemporary*  *Fuels* | Natural gas | 40 (28, 58) | 255 |
|  | Electricity | 39 (31, 48) | 50.4 (10.2, 272) |
|  | Propane | 3 (1, 7) | 300 |
|  | Heating oil | 0.03 (0, 17) | 361 |
|  | Coal or Coke | 4^a^ | 438 |
|  | Non-fossil | 8 (3 to 16) | 0^b^ |
| *Future Fuels* | Natural gas | 51.9 | 255 |
|  | Electricity | 30.7 | 10.2 (10.1, 18.9) |
|  | Heating oil | 3.4 | 361 |
|  | Non-fossil | 13.9 | 0^b^ |

^a^ Only 7 TSAs had non-zero coke or coal and their average value was 4%.

^b^ Non-fossil emissions were assumed to be 0 in the optimization to maximize avoided emissions.

# *Restricted Harvest* Age Thresholds

The *Restricted Harvest* scenario defined harvest age thresholds based on natural disturbance type (Figure S2, Table S5).


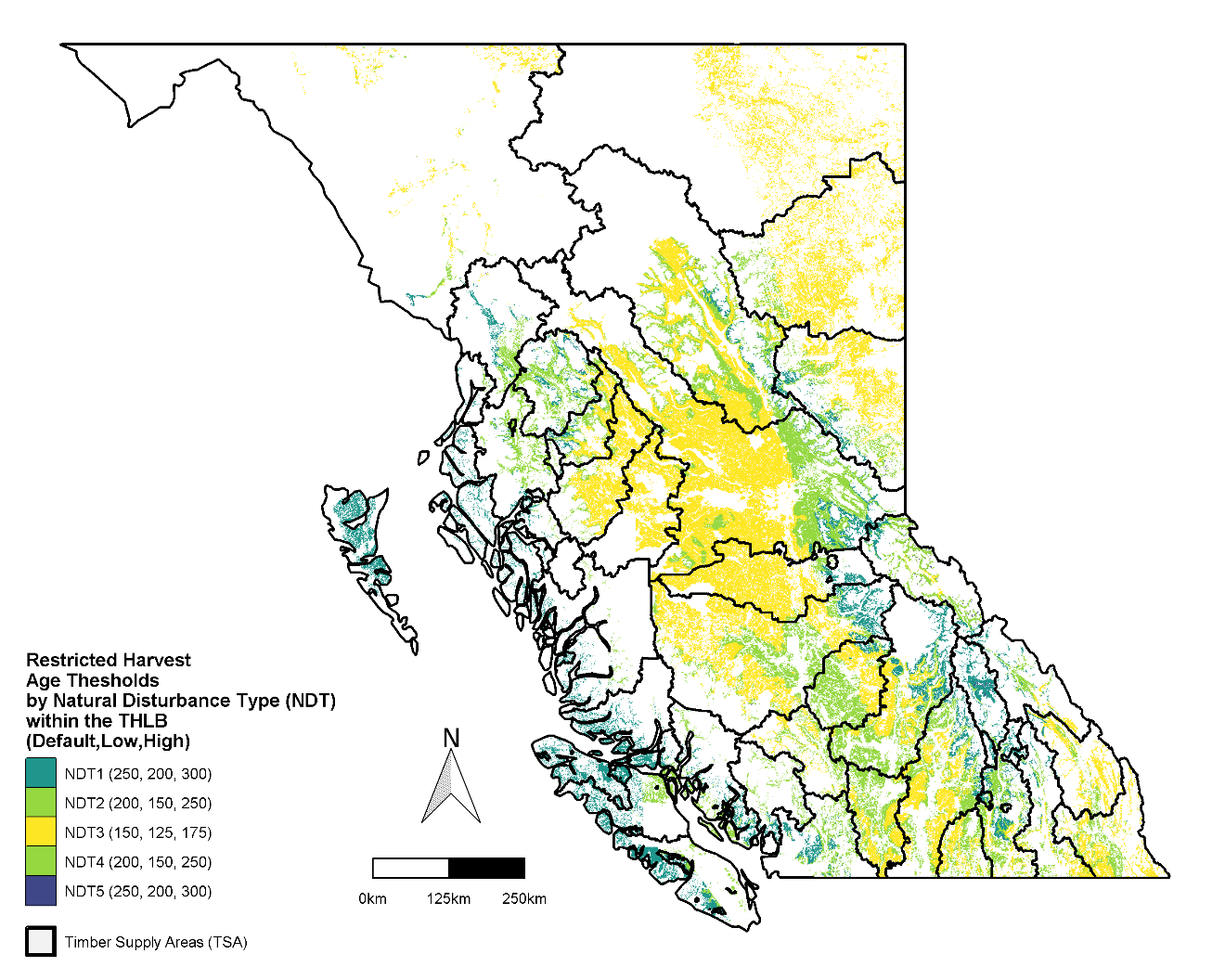


*Figure S2. Restricted Harvest age thresholds within the THLB based on natural disturbance types.*

The province of British Columbia developed natural disturbance classifications based on each ecosystem’s natural disturbance regime, and we subsequently used the disturbance regime to develop old-growth age thresholds for each NDT and associated BEC sub-zones (BC Ministry of Forests and BC Environment 1995). These age thresholds were applied to aggregated forest types (deciduous and conifer stands) and updated with newer information and: old-growth age thresholds for the Interior Douglas Fir (IDF) zone was reduced to 125 years to account for the shorter return intervals identified (Wong et al. 2003), and NDT2 ages were reduced from 250 to 200 years (Franklin et al. 2002; Wells et al. 1998). NDT5 did not have an old-growth age threshold assigned in the original natural disturbance classification because there was a lack of disturbance research in the Alpine Tundra (AT) zone, but we assumed NDT1 values because the disturbance return intervals were similar (Wong et al., 2003).

*Table S5. Age thresholds for the Restricted Harvest scenario based on the BEC zone, subzone, and Natural Disturbance Type (NDT).*

| **Age threshold (years)**  **Default (high, low) implementation level** | **BEC zone** | **Subzone** | **Code** | **NDT** |
| --- | --- | --- | --- | --- |
| 250 (200, 300) | **Coastal Western Hemlock** |  | **CWH** |  |
|  |  | Undifferentiated | un | 1 |
|  |  | Very Wet Hypermaritime | Vh | 1 |
|  |  | Very Wet Maritime | Vm | 1 |
|  |  | Wet Hypermaritime | Wh | 1 |
|  |  | Wet Maritime | Wm | 1 |
|  | **Engelmann Spruce -- Subalpine Fir** |  | **ESSF** |  |
|  |  | Very Wet Cold | vc | 1 |
|  |  | Very Wet Cold Woodland | vcw | 1 |
|  |  | Wet Cold | wc | 1 |
|  |  | Wet Cold Woodland | wcw | 1 |
|  |  | Wet Hot | wh | 1 |
|  |  | Wet Cool | wk | 1 |
|  |  | Wet Mild | wm | 1 |
|  |  | Wet Mild Woodland | wmw | 1 |
|  |  | Wet Very Cold | wv | 1 |
|  | **Mountain Hemlock** |  | **MH** |  |
|  |  | Moist Maritime | mm | 1 |
|  |  | Undifferentiated | un | 1 |
|  |  | Wet Hypermaritime | wh | 1 |
|  | **Coastal Mountain-heather Alpine** |  | **CMA** |  |
|  |  | Undifferentiated | un | 5 |
|  |  | Undifferentiated and Parkland | unp | 5 |
|  |  | Wet Hypermaritime | wh | 1 |
|  | **Boreal Altai Fescue Alpine** |  | **BAFA** |  |
|  |  | Undifferentiated | un | 5 |
|  |  | Undifferentiated and Parkland | unp | 5 |
|  | **Interior Cedar – Hemlock** |  | **ICH** |  |
|  |  | Very Wet Cold | vc | 1 |
|  |  | Very Wet Cool | vk | 1 |
|  |  | Shuswap Wet Cool | wk | 1 |
| 200 (150, 250) | **Coastal Western Hemlock** |  | **CWH** |  |
|  |  | Dry Maritime | dm | 2 |
|  |  | Dry Submaritime | ds | 2 |
|  |  | Submontane Moist Maritime | mm | 2 |
|  |  | Central Moist Submaritime | ms | 2 |
|  |  | Submontane Wet Submaritime | ws | 2 |
|  |  | Very Dry Maritime | xm | 2 |
|  |  | Moist Submaritime | ms | 2 |
|  | **Coastal Douglas-fir** |  | **CDF** |  |
|  |  | Moist Maritime | mm | 2 |
|  | **Engelmann Spruce -- Subalpine Fir** |  | **ESSF** |  |
|  |  | Dry Cold | dc | 2 |
|  |  | Dry Cold Woodland | dcw | 2 |
|  |  | Moist Hot | mh | 2 |
|  |  | Wet Hot | wh | 2 |
|  |  | Ymir Wet Mild | wm | 2 |
|  |  | Moist Mild Woodland | mmw | 2 |
|  |  | Moist Mild | mm | 2 |
|  |  | Moist Warm | mw | 2 |
|  |  | Moist Warm Woodland | mww | 2 |
|  |  | Moist Cool | mk | 2 |
|  |  | Moist Very Cold | mv | 2 |
|  |  | Moist Cold | mc | 2 |
|  |  | Moist Very Cold | mv | 2 |
|  |  | Very Dry Very Cold | xv | 2 |
|  | **Interior Cedar – Hemlock** |  | **ICH** |  |
|  |  | Moist Cold | mc | 2 |
|  |  | Moist Cool | mk | 2 |
|  |  | Moist Mild | mm | 2 |
|  |  | Moist Warm | mw | 2 |
|  |  | Wet Cold | wc | 2 |
|  |  | Very Dry Warm | xw | 4 |
|  | **Sub-Boreal Spruce** |  | **SBS** |  |
|  |  | Wet Cool | wk | 2 |
|  |  | Very Wet Cool | vk | 2 |
|  | **Spruce – Willow -- Birch** |  | **SWB** |  |
|  |  | Undifferentiated | un | 2 |
|  |  | Undifferentiated Scrub | uns | 2 |
|  |  | Moist Cool | mk | 2 |
|  |  | Moist Cool Scrub | mks | 2 |
|  |  | Very Wet Cool | vk | 2 |
|  |  | Very Wet Cool Scrub | vks | 2 |
|  | **Bunchgrass** |  | **BB** |  |
|  |  | Very Dry Hot | xh1 | 4 |
|  |  | Very Dry Warm | xw | 4 |
|  | **Interior Douglas-fir** |  | **IDF** |  |
|  |  | Dry Cold | dc | 4 |
|  |  | Dry Cool | dk | 4 |
|  |  | Dry Mild | dm | 4 |
|  |  | Dry Warm | dw | 4 |
|  |  | Moist Warm | mw | 4 |
|  |  | Wet Warm | ww | 4 |
|  |  | Very Dry Cold | xc | 4 |
|  |  | Very Dry Hot | xh | 4 |
|  |  | Very Dry Cool | xk | 4 |
|  |  | Very Dry Mild | xm | 4 |
|  |  | Very Dry Warm | xw | 4 |
|  | **Ponderosa Pine** |  | **PP** |  |
|  |  | Dry Hot | dh | 4 |
|  |  | Very Dry Hot | xh | 4 |
| 150 (125, 175) | **BWBS** |  | **BWBS** |  |
|  |  | Dry Cool | dk | 3 |
|  |  | Moist Cool | mk | 3 |
|  |  | Moist Warm | mw | 3 |
|  |  | Very Wet Cool | vk | 3 |
|  |  | Wet Cool | wk | 3 |
|  | **Engelmann Spruce – Subalpine Fir** |  | **ESSF** |  |
|  |  | Dry Cool Woodland | dkw | 3 |
|  |  | Undifferentiated | un | 3 |
|  |  | Dry Cool | dk | 3 |
|  |  | Dry Cool Woodland | dcw | 3 |
|  |  | Dry Cold | dc | 3 |
|  |  | Dry Very Cold | dv | 3 |
|  |  | Very Dry Cold | xc | 3 |
|  |  | Dry Very Cold Woodland | dvw | 3 |
|  |  | Very Dry Cold Woodland | xcw | 3 |
|  | **Interior Cedar – Hemlock** |  | **ICH** |  |
|  |  | Dry Cool | dk | 3 |
|  |  | Dry Mild | dm | 3 |
|  |  | Dry Warm | dw | 3 |
|  |  | Moist Cool | mk 1 | 3 |
|  | **Montane Spruce** |  | **MS** |  |
|  |  | Dry Cold | dc | 3 |
|  |  | Dry Cool | dk | 3 |
|  |  | Dry Mild | dm | 3 |
|  |  | Dry Very Cold | dv | 3 |
|  |  | Moist Warm | mw | 3 |
|  |  | Undifferentiated | un | 3 |
|  |  | Very Dry Cool | xk | 3 |
|  |  | Very Dry Very Cold | xv | 3 |
|  | **Sub-Boreal Spruce** |  | **SBS** |  |
|  |  | Dry Hot | dh | 3 |
|  |  | Dry Cool | dk | 3 |
|  |  | Dry Warm | dw | 3 |
|  |  | Moist Cold | mc | 3 |
|  |  | Moist Cool | mk | 3 |
|  |  | Moist Mild | mm | 3 |
|  |  | Moist Warm | mw | 3 |
|  |  | Undifferentiated | un | 3 |
|  |  | Wet Cool | wk | 3 |
|  | **Sub-Boreal Pine – Spruce** |  | **SBPS** |  |
|  |  | Dry Cold | dc | 3 |
|  |  | Moist Cold | mc | 3 |
|  |  | Moist Cool | mk | 3 |
|  |  | Very Dry Cold | xc | 3 |

# Mitigation scenarios

Figure S3 shows summary boxplots of energy displacement factors by implementation level and two baseline fuel assumptions (contemporary, future).


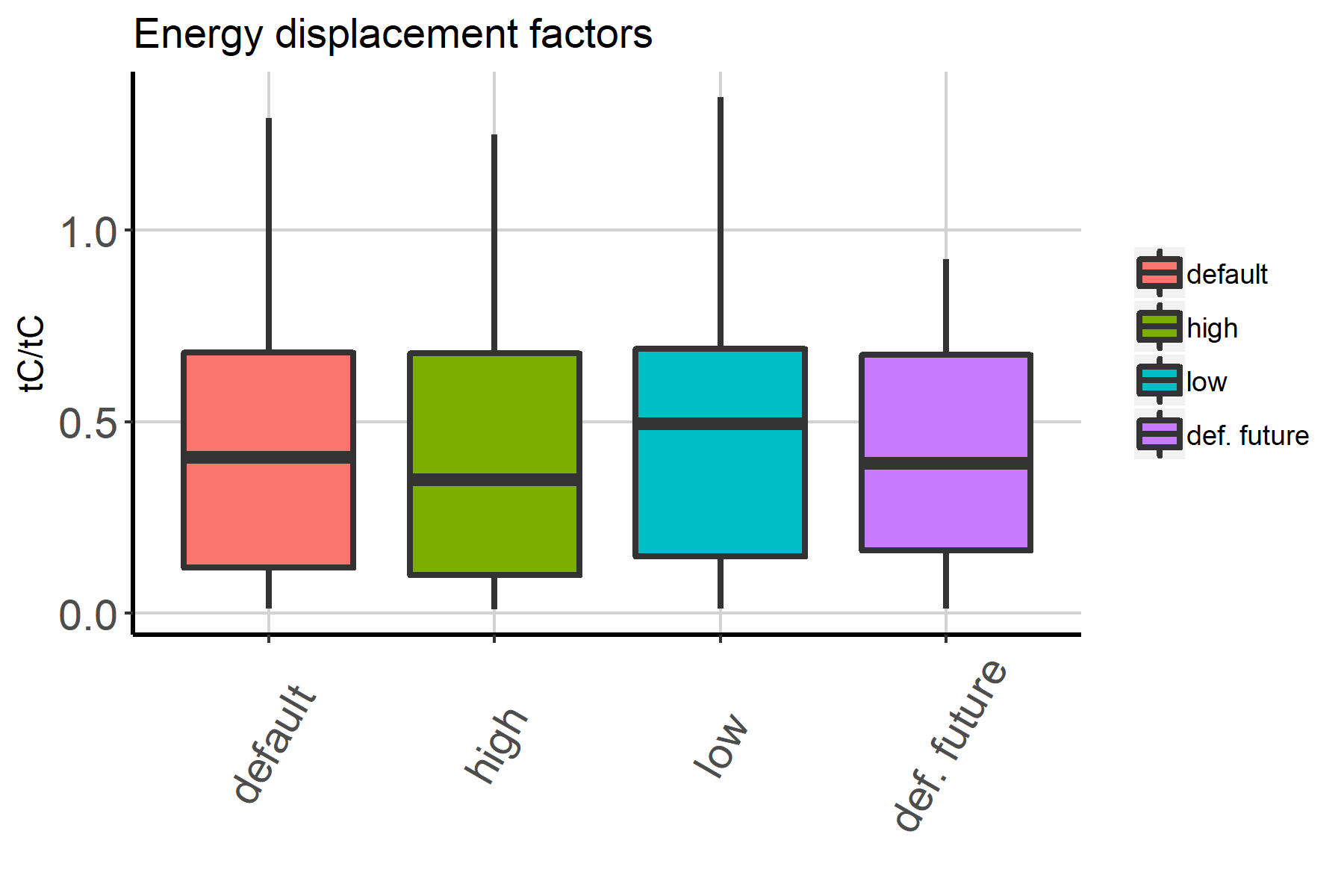


*Figure S3. Boxplots of displacement factors (tC avoided per tC of harvest residues used) for each of the three Harvest Residues for Bioenergy scenario harvest residue capture rates (default, high, and low), assuming baseline community-level energy information, and with baseline future fuels, default scenario implementation level only (def. future).*

*Table S6. Annual average mitigation potential (MtCO_2_e yr^-1^) for portfolios by decade for a) low and b) high scenario implementation levels and high substitution benefits.*

1. Low implementation level

|  | Short-term 2030 portfolio | | Mid-term 2050 portfolio | | Long-term 2070 portfolio | |
| --- | --- | --- | --- | --- | --- | --- |
|  | Global | Domestic | Global | Domestic | Global | Domestic |
| 2020-2029 | -9.0 | -6.2 | -9.0 | -6.2 | -8.8 | -6.0 |
| 2030-2039 | -7.6 | -5.7 | -7.6 | -5.8 | -7.6 | -5.9 |
| 2040-2049 | -7.2 | -5.3 | -7.2 | -5.5 | -7.3 | -5.7 |
| 2050-2059 | -7.2 | -5.3 | -7.2 | -5.6 | -7.5 | -5.6 |
| 2060-2069 | -7.6 | -5.6 | -7.6 | -5.8 | -7.8 | -5.8 |
| Total | -397 | -288 | -397 | -296 | -400 | -296 |

1. High implementation level

|  | Short-term 2030 portfolio | | Mid-term 2050 portfolio | | Long-term 2070 portfolio | |
| --- | --- | --- | --- | --- | --- | --- |
|  | Global | Domestic | Global | Domestic | Global | Domestic |
| 2020-2029 | -13.8 | -9.1 | -13.1 | -8.3 | -11.7 | -8.0 |
| 2030-2039 | -13.4 | -11.9 | -13.8 | -13.1 | -13.8 | -13.2 |
| 2040-2049 | -13.3 | -13.1 | -14.2 | -15.7 | -14.9 | -15.8 |
| 2050-2059 | -13.4 | -13.6 | -14.5 | -16.3 | -15.7 | -16.6 |
| 2060-2069 | -13.8 | -13.7 | -15.1 | -15.9 | -16.6 | -16.3 |
| Total | -690 | -620 | -720 | -697 | -736 | -702 |

1. Global - High b) Domestic - High


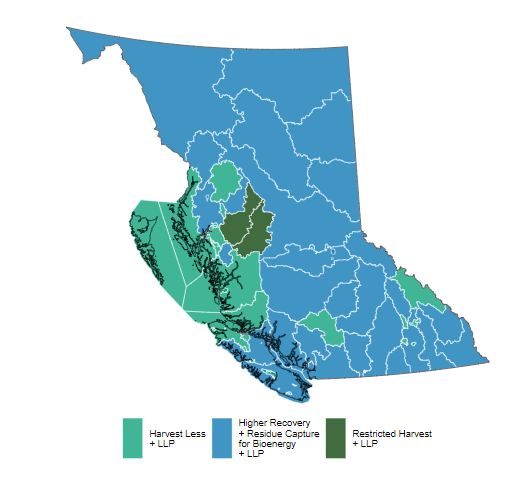

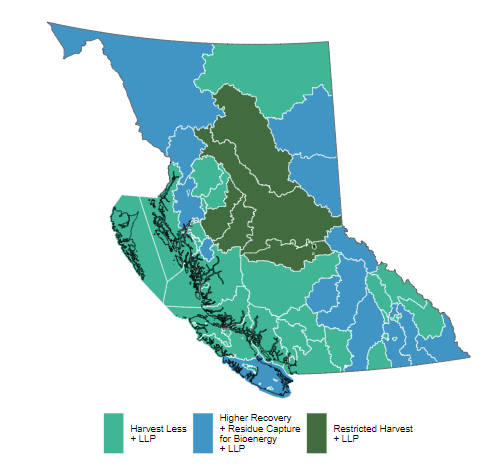


1. Global - Low d) Domestic – Low


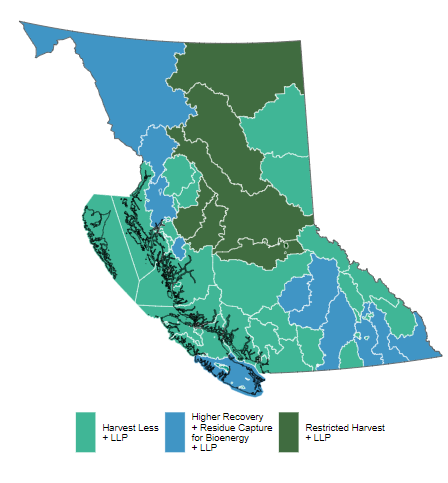

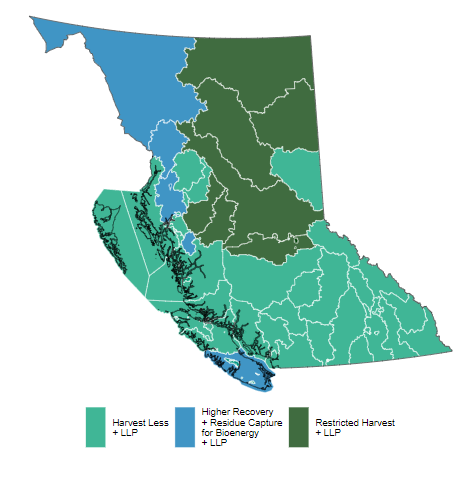


*Figure S4. Scenarios selected for the 2070 global and domestic portfolio, default implementation level, high substitution benefits (a and b) and low substitution benefits (c and d).*

*Table S7. Coefficients from the linear regression of the cumulative 2070 net GHG emissions in log base 10 versus the log base 10 magnitude of the change in harvest (roundwood and harvest residues), all implementation levels. Normalized mitigation potentials are indicated by the non-dimensional slope coefficient.*

| Substitution Benefits | Scenario | Slope | Intercept  (log_10_ MtCO_2_e) | r^2^ | 2070 Cumulative mitigation potential by  implementation level  (MtCO2e) | | |
| --- | --- | --- | --- | --- | --- | --- | --- |
|  |  |  |  |  | Default | High | Low |
| High | *Bioenergy + LLP* | -1.18 | 0.27 | 0.73 | 372 | 450 | 293 |
| High | *Higher Recovery + Bioenergy* | -1.17 | 0.45 | 0.65 | 322 | 379 | 285 |
| Low | *Bioenergy + LLP* | -1.16 | 0.53 | 0.39 | 221 | 234 | 205 |
| High | *Higher Recovery + Bioenergy + LLP* | -1.1 | 0.13 | 0.85 | 527 | 686 | 395 |
| High | *Restricted Harvest* | -1.06 | 0 | 0.96 | 116 | 216 | 45 |
| Low | *Restricted Harvest* | -1.04 | -0.26 | 0.99 | 208 | 374 | 85 |
| Low | *Higher Recovery* | -1.03 | 0.71 | 0.97 | 25 | 36 | 17 |
| Low | *Higher Recovery + Bioenergy + LLP* | -1.03 | 0.37 | 0.58 | 268 | 307 | 236 |
| High | *Harvest Residues for Bioenergy* | -1.02 | 0.42 | 0.41 | 191 | 189 | 193 |
| High | *Higher Recovery* | -1.01 | -0.01 | 1 | 123 | 181 | 84 |
| Low | *Harvest Less* | -0.99 | -0.35 | 0.99 | 300 | 602 | 60 |
| High | *Harvest Less* | -0.98 | -0.16 | 0.98 | 187 | 373 | 37 |
| High | *Higher Recovery + LLP* | -0.92 | -0.48 | 0.94 | 328 | 488 | 195 |
| Low | *Higher Recovery + LLP* | -0.9 | 0.03 | 0.9 | 101 | 150 | 58 |
| Low | *Harvest Less + LLP* | -0.89 | -0.50 | 0.99 | 362 | 682 | 97 |
| High | *Harvest Less + LLP* | -0.76 | -0.57 | 0.93 | 355 | 590 | 137 |
| Low | *Higher Recovery + Bioenergy* | -0.76 | 0.19 | 0.32 | 194 | 197 | 196 |
| Low | *Harvest Residues for Bioenergy* | -0.75 | 0.19 | 0.25 | 156 | 143 | 169 |
| Low | *Restricted Harvest + LLP* | -0.75 | -0.56 | 0.93 | 270 | 434 | 138 |
| High | *Restricted Harvest + LLP* | -0.54 | -0.70 | 0.78 | 283 | 376 | 188 |


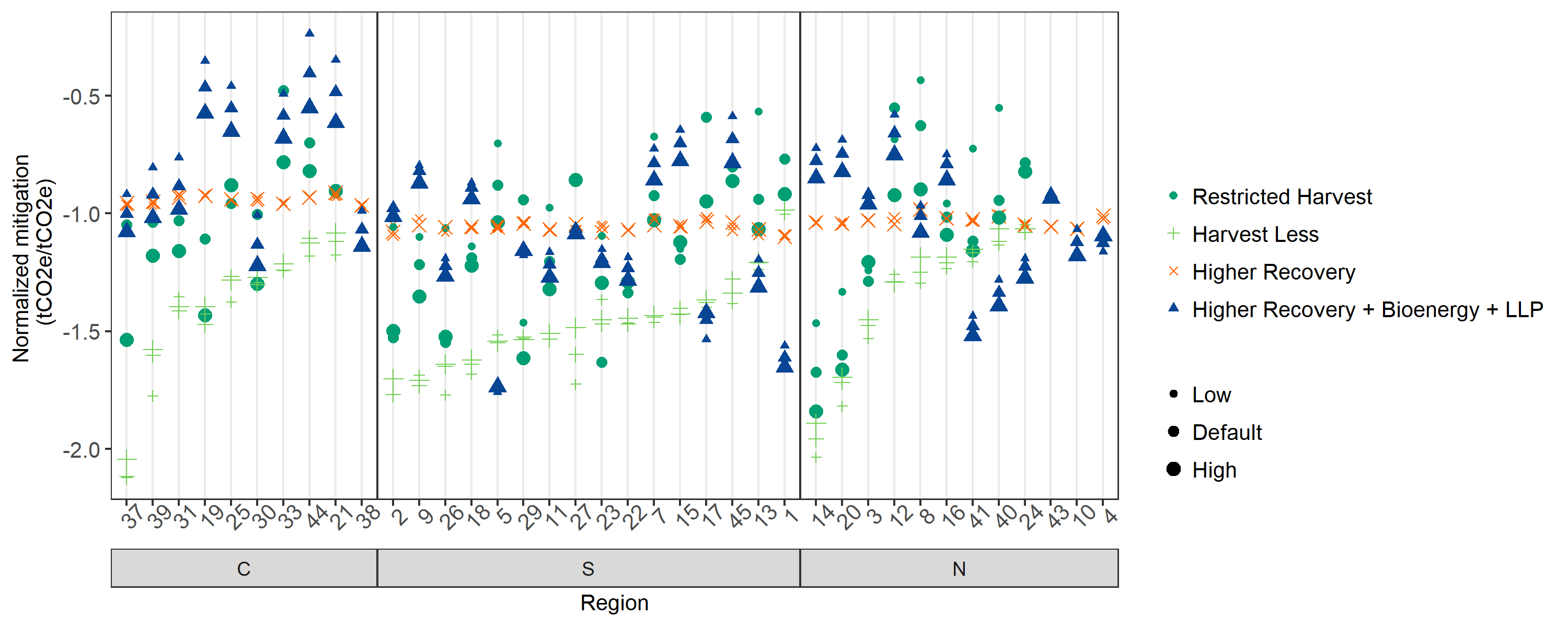


*Figure S5. Ratio of the 2070 cumulative mitigation to cumulative harvest difference (magnitude of change in roundwood and harvest residues) for each scenario and Timber Supply Area. Results are grouped by region: C=Coast, S=South and N=North. Symbol sizes reflect the three implementation levels.*

# Future Fire Risk: Monte Carlo Fires

Fire layers from generated using methods by Metsaranta *et al.* (2011) and updated with recent data were used to assess risk of reversal for conservation scenarios. Briefly, historical timeseries of area burned and number of fires from 1950 to 2018 were fitted to log-normal distributions for three landscapes: coast, southern interior, and northern interior. Timeseries from 2020 to 2070 area burned were constructed from these distributions with the assumption that the annual area burned would linearly increase and result in a doubling of the area burned in 2070. Total annual burned area and number of fires were drawn 100 times and resulting spatial fire layers were constructed assuming ellipsoidal fires that were randomly placed on publically-owned forests. Two thresholds were applied to the draws: minimum fire size was assumed to be 100 ha; and maximum area burned could not exceed twice the historical maximum multiplied by the linear increase (doubled area over 50 year). We further assumed that a stand could not reburn for at least 10 years. Average overlap percentages of the cumulative conserved stands that subsequently burned for the three regions were generally less than 1% (Figure S6). Average uncertainty ranges based on the difference between the 2.5 and 97.5 percentiles were higher: 0.63% for the coastal region, 2.7% for the northern interior and 2.6% for the southern interior. Annual uncertainty ranges had a maximum of 14% for the southern interior, indicating that 14% of all conserved stands could burn in one year for that region.

*Figure S6. Overlap percentage of Monte Carlo fire layers with the cumulative conserved stands in the Harvest Less scenario (default implementation). Average annual overlap percentages (solid lines) were used to assess an ex-post netdown for the forest mitigation component. Uncertainty ranges based on the 95% confidence interval (dotted lines) are shown up to 6%, but the maximum value was 14%.*

*Table S8. Percentage reduction in cumulative mitigation potential in 2070 for default implementation levels of the Harvest Less and Restricted Harvest scenario, based on the average cumulative area overlap between conserved stands and 100 future fire draws.*

|  | Percentage reduction in cumulative mitigation potential (%) | | | |
| --- | --- | --- | --- | --- |
| Scenario | Coast | South | North | Total |
| *Harvest Less* | 3.4 | 12.5 | 15.1 | 12.1 |
| *Restricted Harvest* | 3.9 | 12.0 | 15.5 | 14.2 |

*Figure S7. Timeseries of the deadwood density.*


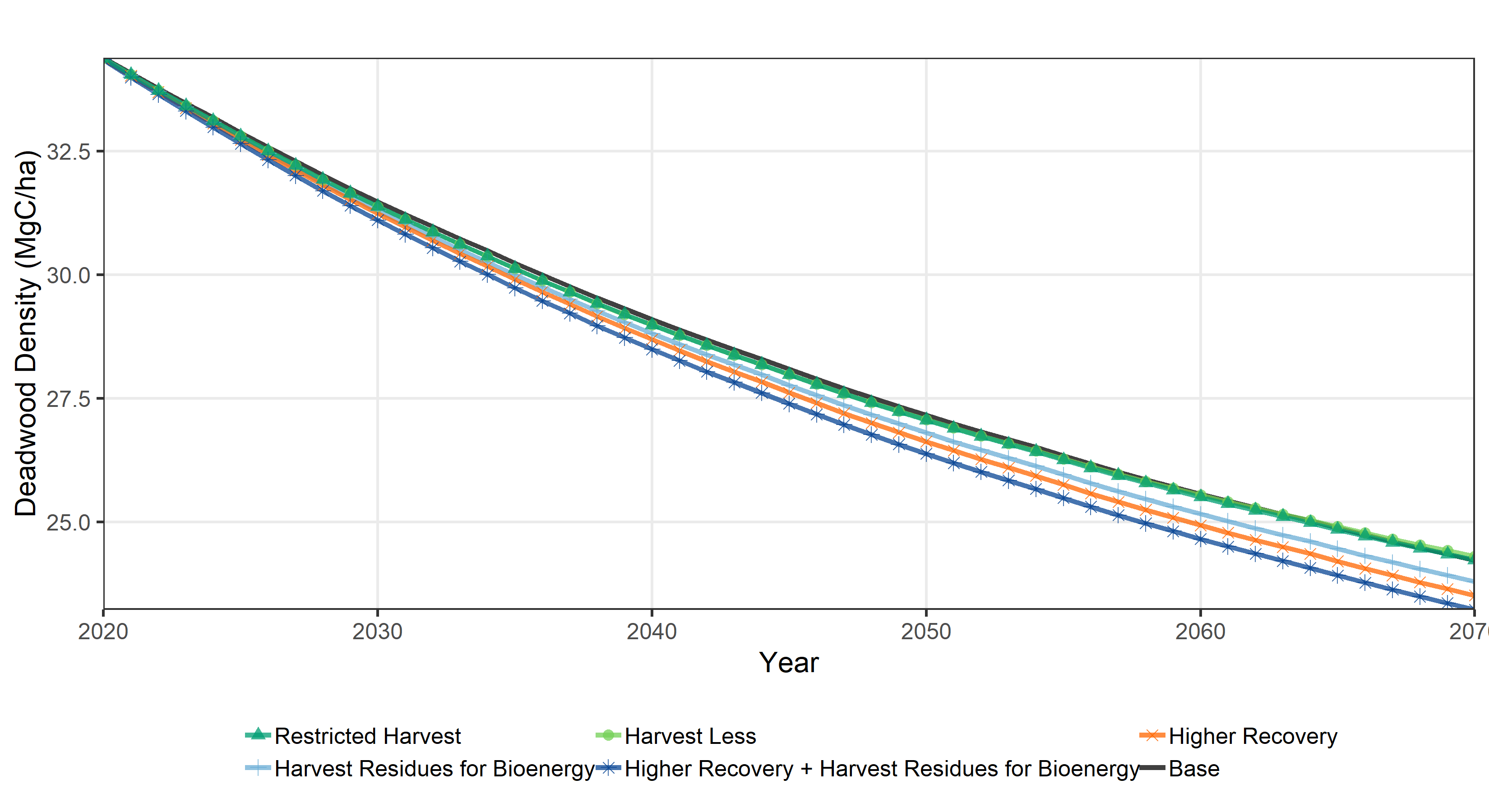


*Figure S8. Future timber supply components a) net merchantable growth and b) harvest transfer to HWP.*

a)


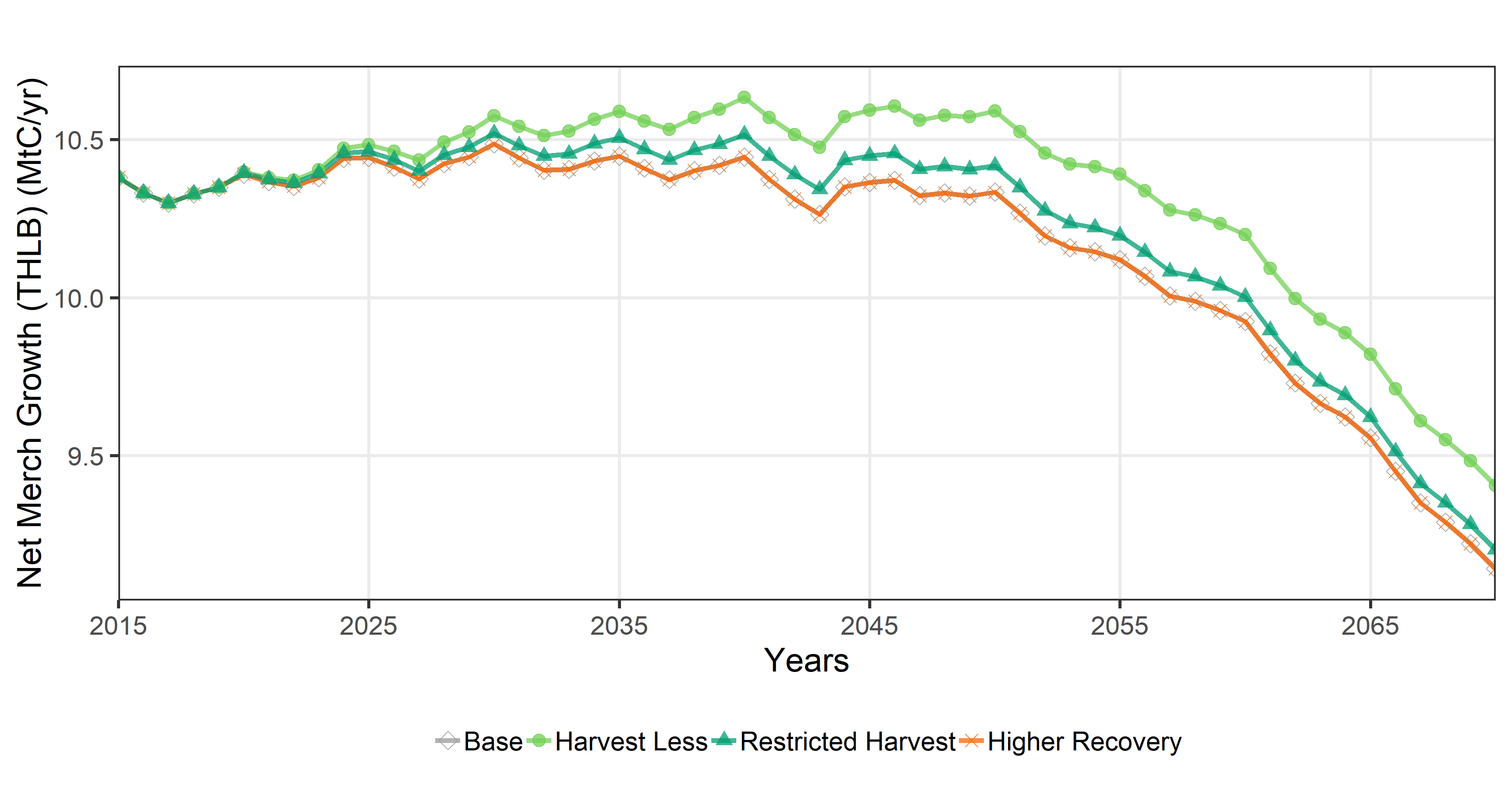


b)


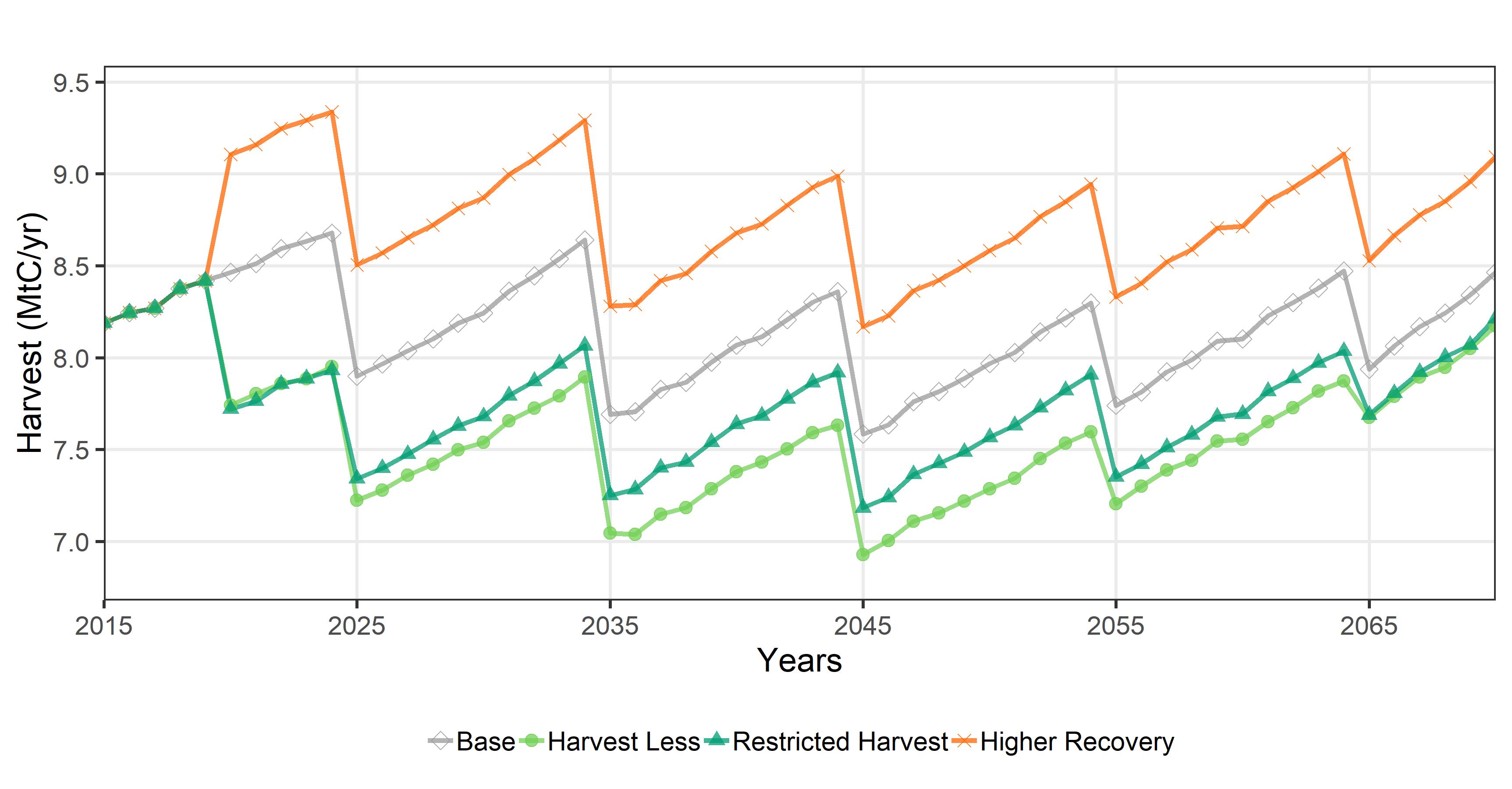


# Economic Prices and Costs

For the prices and costs for harvesting and products, we used annual averages to reflect long-term trends and assumed that they did not change over time. Softwood and hardwood log costs include tree-to-truck cost, hauling cost, cost of stumpage, and costs for forest planning and administration, road development and management, and silviculture. Softwood and hardwood log prices for mitigation scenarios were assumed to change because of changing log quality. Harvest residues has no baseline price because no opportunity cost was assumed.

*Table S9. Harvest cost and price assumptions for the baseline and mitigation scenarios (2018 dollars).*

| Scenario | Forest Region | Softwood Log Price ($/m^3^)*^a^* | Hardwood Log Price ($/m^3^)*^b^* | Salvage Log Price ($/m^3^)*^c^* | Harvest Residue Price ($/tC) | Softwood Log Cost ($/m^3^)*^d^* | Hardwood Log Cost ($/m^3^)*^b^* | Salvage Log Cost ($/m^3^)*^e^* | Harvest Residue Cost ($/tC)*^f^* |
| --- | --- | --- | --- | --- | --- | --- | --- | --- | --- |
| Baseline*^g^* | Northern Interior*^h^* | 71.34 | 61.34 | 36.05 | 0 | 62.64 | 52.64 | 31.05 | 15.16 |
|  | Southern Interior | 77.04 | 67.04 | 38.93 | 0 | 67.65 | 57.65 | 33.93 | 15.16 |
|  | Coast | 129.86 | 119.86 | 62.52 | 0 | 101.54 | 91.54 | 57.52 | 15.16 |
| Baseline (4 TSAs with low utilization)*^i^* | Kalum/Cassiar | 77.68 | 67.68 | 36.05 | 0 | 68.21 | 58.09 | 31.05 | 15.16 |
|  | Nass | 128.43 | 118.43 | 38.93 | 0 | 112.78 | 101.84 | 33.93 | 15.16 |
|  | Arrowsmith | 131.96 | 121.96 | 62.52 | 0 | 103.18 | 93.14 | 57.52 | 15.16 |
| Higher Recovery | Interior | 1.85 (1.13, 2.86) decrease*^j^* | | No change | | 0.09 (0.05, 0.14) decrease*^k^* | | No change | |
|  | Coast | 4.92 (3.02, 7.62) decrease | |  |  | 0.17 (0.1, 0.26) decrease | |  |  |
| Higher Recovery (4 TSAs) | Kalum | 5.91 (5.29, 6.79) decrease | | No change | | 0.28 (0.25, 0.33) decrease | | No change | |
|  | Cassiar | 4.43 (3.78, 5.36) decrease | |  |  | 0.21 (0.18, 0.26) decrease | |  |  |
|  | Nass | 23.26 (23.04, 23.59) decrease | |  |  | 1.11 (1.07, 1.16) decrease | |  |  |
|  | Arrowsmith | 19.68 (18.11, 21.9) decrease | |  |  | 0.66 (0.60, 0.76) decrease | |  |  |
| Harvest Less*^l^* | Interior/Coast | No change | | | | 0.69 (0.34) increase | | No change | |
| Harvest Residues for Bioenergy | Interior | No change | | | | 0.18 decrease | | No change | 17.54*^m^* |
|  | Coast |  |  |  |  |  |  |  | 16.33*^m^* |
| Restricted Harvest | Northern Interior | 0.33 (0.15, 0.57) decrease | | No change | | 0.88 (0.7, 1.15) increase | | No change | |
|  | Southern Interior | 0.21 (0.07, 0.49) decrease | |  |  |  |  |  |  |
|  | Coast | 2.17 (0.18, 8.36) decrease | |  |  | 1.11 (1.08, 1.22) increase | |  |  |

*^a^* Log Market Reports (2014-2018 averages), BCMoFLNRO. <http://www2.gov.bc.ca/gov/content/industry/forestry/competitive-forest-industry/timber-pricing>

*^b^* Assuming $10/m^3^ less than the softwood log prices, personal communications with BCMoFLNRO.

*^c^* Log Market Reports (2014-2018 averages), pulplog prices for interior and hembal prices for coast were used.

*^d^* Personal communication with BCMoFLNRO.

*^e^* Assumed to be $5/m^3^ lower than salvage log prices, personal communication with BCMoFLNRO.

*^f^* Including a $5.58/tCO_2_e slashburing cost and a $50/tCO_2_e GHG emission penalty.

*^g^* Assumptions for TSAs with the default 85% harvest utilization level in the baseline.

*^h^* Log prices in northern interior was assumed to be 8% lower than southern interior, according to personal communication with BCMoFLNRO.

*^i^* Assumptions for the four TSAs with lower than 85% utilization levels only including species that provide more valuable logs. Those TSAs were not included in other scenarios.

*^j^* Values in brackets are for the low and high implementation levels, respectively. Price changes for all scenarios were calculated based on changes in proportions of different log grades.

*^k^* Personal communication with FPInnovations for cost changes in all scenarios.

*^l^* No assumptions for high implementation level.

*^m^* Calculated based on the processing and other costs in Table S14.

Harvests were used to produce generic HWP commodities: sawnwood, other industrial roundwood, panels, and pulp and paper products which replaced more GHG intensive materials like concrete and plastic (no steel was assumed to be produced in BC). The associated price and cost assumptions are shown in Tables S10 to S12. Note that the delivered fiber cost has been removed from the cost and price assumptions in Tables S10 and S11 for both baseline and mitigation scenarios to avoid double counting. It has been already covered in the log price assumptions in Table S9.

*Table S10. Price assumptions for harvested wood products in all scenarios (2018 dollars).*

| Forest Region | Sawnwood Price ($/m^3^)*^a^* | Panel Price ($/m^3^)*^a^* | Other Industrial Roundwood Price ($/m^3^)*^b^* | Pulp Price ($/odt)*^c^* |
| --- | --- | --- | --- | --- |
| Northern Interior | 80 | 196 | 80 | 613 |
| Southern Interior | 80 | 196 | 80 | 613 |
| Coast | 80 | 196 | 80 | 613 |

*^a^* Prices were calculated based on manufacturing costs and cost-price ratios for typical products using 2010-2017 averages from Forest Economic Advisors LLC.

*^b^* Assumed to be the same as sawnwood prices.

*^c^* Market Pulp 2013-2018 averages from PulpMarket.

*Table S11. Cost assumptions for harvested wood products in the baseline and mitigation scenarios (2018 dollars). Values in brackets are for the low and high implementation levels, respectively.*

| Scenario | Forest Region | Sawnwood Cost ($/m^3^)*^a^* | Panel Cost ($/m^3^)*^b^* | Other Industrial Roundwood Cost ($/m^3^)*^c^* | Pulp Cost ($/odt)*^d^* |
| --- | --- | --- | --- | --- | --- |
| Baseline | Northern Interior | 64 | 180 | 64 | 450 |
|  | Southern Interior | 64 | 180 | 64 | 481 |
|  | Coast | 64 | 180 | 64 | 441 |
| Higher Recovery | Interior/Coast | No change*^e^* | | | 97.5% (98.5%, 96%) of the baseline assumptions*^f^* |
| Harvest Less | Interior/Coast | 105% (101%) of the baseline assumptions*^f^* | | | |
| Harvest Residues for Bioenergy | Interior/Coast | No change | | | |
| Restricted Harvest | Northern Interior | 104% (102%, 107%) of the baseline assumptions*^f^* | | | |
|  | Southern Interior | 103% (101%, 106%) of the baseline assumptions*^f^* | | | |
|  | Coast | 101% (100%, 102%) of the baseline assumptions*^f^* | | | |
| More Longer-lived Products | Interior/Coast | 98.5% (99.25%, 97.75%) of the baseline assumptions*^f^* | | No change | 103% (101.5%, 104.5%) of the baseline assumptions*^f^* |

*^a^* Average manufacturing cost for lumber in BC during 2010-2017, Forest Economic Advisors LLC.

*^b^* Average manufacturing cost for OSB (3/8 inch), particleboard and plywood in Western Canada during 2010-2017, Forest Economic Advisors LLC.

*^c^* Assumed to be the same as sawnwood prices.

*^d^* Average cost (manufacturing plus transportation) for NBSK in 2012 and 2014, FisherSolve, Fisher International Inc.

*^e^* Higher manufacturing cost due to lower log quality was assumed to be captured by the decreased log prices to avoid double counting.

*^f^* Based on the ratio of changes in the harvest amount and manufacturing cost in Table 5 in Xu et al. (2017) to reflect the impacts of manufacturing efficiency.

*Table S12. Cost and price assumptions for substituted products in strategies involving substitution benefits (2018 dollars)^a^.*

| Concrete Price ($/tonne)*^b^* | Plastic Price ($/tonne)*^c^* | Concrete Cost ($/tonne)*^b^* | Plastic Cost ($/tonne)*^c^* |
| --- | --- | --- | --- |
| 75.15 | 1988.80 | 73.99 | 1878.06 |

*^a^* Costs and Prices were assumed unchanged across scenarios.

*^b^* NRMCA (2012)

*^c^* Market price and manufacturing cost for virgin HDPE plastic, sourced from <http://www.wrap.org.uk/content/plastic> and converted to CAD 2018 dollars.

Prices and costs for bioenergy were determined by the results of the Linear Programming (LP) model that selected the number and type of facilities for each region from nine candidate facilities (Table S13). Since we assumed that there was no bioenergy production from harvest residues in the baseline, no baseline prices and costs were needed. In the bioenergy strategy, the price of bioenergy in each region was calculated based on generic electricity price ($120/MWh) and heat price ($8/GJ) weighted by proportions of power and heat that bioenergy generated. The bioenergy production cost in the bioenergy strategy was estimated by the optimization model selected facilities and their associated production costs. The bioenergy production cost also included costs for transporting harvest residues to facilities based on simplified transport distance assumptions. No cost was assumed for extracting harvest residue from cut blocks to roadside, because the full-tree harvesting approach was assumed to be employed in BC. The prices for fossil fuel energy in the bioenergy strategy were the same as the prices for bioenergy, while the unit costs were calculated by dividing the total energy production cost (including fuel cost and production cost) from all fuel sources being substituted by the total bioenergy production (Table S15).

*Table S13. Bioenergy facility types and characteristics adapted from Smyth et al. (2017).*

| Type | Scale | Description | Biomass demand  (kodt/yr) | Electrical conversion  rate  (MWh/odt) | Thermal conversion  rate  (GJ/odt) | Assumed electrical efficiency  (%) | Assumed thermal efficiency  (%) | Implied  overall efficiency  (%) | Production Cost*^f^* ($/MWh) |
| --- | --- | --- | --- | --- | --- | --- | --- | --- | --- |
| Heat | Small | 0.4 MWth boiler for district heating*^a^* | 0.783 | - | 15.0 | - | 75 | 75 | 13.28 |
|  | Medium | 2.3 MWth boiler for district heating*^a^* | 3.97 | - | 17.0 | - | 85 | 85 | 9.99 |
|  | Large | 6.62 MWth process heat via syngas*^b^* | 11.58 | - | 16.8 | - | 84 | 84 | 7.12 |
| Power | Small | 0.2 MWe gas turbine*^c^* | 1.60 | 1.02 | - | 18 | - | 18 | 173.09 |
|  | Medium | 5 MWe steam cycle*^b^* | 34.97 | 1.17 | - | 21 | - | 21 | 34.70 |
|  | Large | 10 MWe steam cycle*^b^* | 63.86 | 1.28 | - | 23 | - | 23 | 30.15 |
| CHP | Small | 0.2 Mwe, 0.98 MWth organic rankine cycle*^d^* | 2.09 | 0.78 | 14.0 | 14 | 70 | 84 | 123.17 |
|  | Medium | 1.8 MWe,4.5MWth steam turbine*^e^* | 10.58 | 1.39 | 10.8 | 25 | 54 | 79 | 63.36 |
|  | Large | 8 MWe CHP steam turbine*^b^* | 46.87 | 1.39 | 5.88 | 25 | 29 | 54 | 56.43 |

*^a^* RETScreen International (2015).

*^b^* Biopathways (FPAC & FPInnovations 2011).

*^c^* Arena et al. (2010).

*^d^* Wood and Rowley (2011).

*^e^* Pröll et al. (2011).

*^f^* Production costs (2018 dollars) include fiber costs.

*Table S14: Cost assumptions for the supply of harvest residues ($/odt in 2008 dollars).*

| Forest Region | Processing Cost ($/odt) | Other Costs ($/odt)*^c^* |
| --- | --- | --- |
|  |  |  |
| Northern/Southern Interior | 21*^a^* | 8 |
| Coast | 19*^b^* | 8 |

*^a^* Figure for BC Interior by Friesen (2013).

*^b^* MacDonald (2012).

^c^ Average of (Ralevic 2013; Ralevic et al. 2010; Reynolds et al. 2012; Ryans and Cormier 2009)

*Table S15. Cost assumptions for power and heat production using fossil fuels (2008 dollars).*

| Energy Type | Fuel Type | Total Cost ($/MWh) |
| --- | --- | --- |
| Heat | Natural gas | 22*^a^* |
|  | Electricity | 42*^b^* |
|  | Fuel oil | 101*^c^* |
|  | Waste fuels | 5*^d^* |
| Power | Diesel | 259*^e^* |
|  | Natural gas | 35*^f^* |

*^a^* NEB (2014), Manitoba Hydro (2015), EPA (2013), and IEA (2010).

*^b^* NEB (2014), Manitoba Hydro (2015), USDC (2011), and Hamilton Home Products (2015).

*^c^* NRCan (2015), EPA (US EPA 2013), and IEA (2010).

*^d^* Waste fuels refer to fuels recovered from industrial processes, such as coke, coke oven gas, petroleum coke, and distilled gas. The total cost was estimated based on EIA (2013b), EPA (2013), and IEA (2010).

*^e^* NRCan (2015), Dunn (2011), and Osler (2011).

*^f^* NEB (2014), Manitoba Hydro (2015), and (EIA 2013a).

# Socio-economic Multipliers

*Table S16. Industries identified for the forest (including bioenergy) sector and associated multipliers.*

| Industry | Direct Jobs | Indirect Jobs | Direct GDP | Indirect GDP | Total Govt. Revenue |
| --- | --- | --- | --- | --- | --- |
| Forestry and Logging | 2.57 | 3.08 | 0.34 | 0.28 | 0.05 |
| Harvest Residue Extraction*^a^* | 3.45 | 2.72 | - | - | - |
| Wood Products Manufacture | 3.79 | 2.86 | 0.34 | 0.30 | 0.03 |
| Pulp, Paper and Paperboard Mills | 1.44 | 2.70 | 0.31 | 0.34 | 0.05 |
| Electric Power Generation, Transmission and Distribution | 1.49 | 1.26 | 0.78 | 0.12 | 0.12 |

*^a^* This industry is not specified in NAICS and was assumed to be the averages of the multipliers for ‘Forestry and Logging’ and ‘Truck Transportation’ industries. Only impacts of harvest residue extraction on employment were considered in the socio-economic impacts of the Bioenergy scenario, because costs for extracting harvest residues were included in bioenergy generation, as were the associated socio-economic impacts.

*Table S17. Industry distribution among strategies and labor intensity assumptions.*

| Logging (Person Year/million m^3^) | Harvest Residue Extraction (Person Year/million m^3^) | Wood Manufacture (Person Year/million m^3^) | Pulp and Paper Manufacture (Person Year/million odt) | Bioenergy Generation (Person Year/million m^3^) |
| --- | --- | --- | --- | --- |
| 171 | 126 | 387 | 272 | 44 |

# Mitigation Cost Estimates and Socio-economic Impacts

*Table S18. Cost per tonne estimates ($ tCO_2_e^‑1^ in 2018 Canadian dollars), 2020-2070 by scenario, implementation level and substitution benefit (Low levels for General Use, Future Fuels; High levels for Wood Buildings, Contemporary Fuels).*

|  | Costs | | |
| --- | --- | --- | --- |
| Scenario | Implementation Level | | |
| (level of substitution benefits) | Default | High | Low |
| *Higher Recovery + LLP (low)* | 155 | 141 | 198 |
| *Higher Recovery + LLP (high)* | 61 | 56 | 75 |
| *Harvest Less + LLP (low)* | 30 | N/A | 41 |
| *Harvest Less + LLP (high)* | 30 | N/A | 34 |
| *Bioenergy + LLP (low)* | 131 | 178 | 89 |
| *Bioenergy + LLP (high)* | 105 | 131 | 65 |
| *Higher Recovery + Bioenergy + LLP (low)* | 127 | 174 | 94 |
| *Higher Recovery + Bioenergy + LLP (high)* | 88 | 112 | 63 |
| *Restricted Harvest + LLP (low)* | 37 | 32 | 47 |
| *Restricted Harvest + LLP (high)* | 37 | 34 | 39 |
| *Higher Recovery (low)* | 272 | 239 | 304 |
| *Higher Recovery (high)* | 47 | 43 | 51 |
| *LLP (low)* | 114 | 108 | 82 |
| *LLP (high)* | 56 | 54 | 41 |
| *Harvest Less (low)* | 22 | N/A | 26 |
| *Harvest Less (high)* | 25 | N/A | 30 |
| *Restricted Harvest (low)* | 24 | 25 | 28 |
| *Restricted Harvest (high)* | 29 | 29 | 34 |
| *Bioenergy (high)* | 114 | 158 | 68 |
| *Bioenergy (low)* | 126 | 195 | 78 |
| *Higher Recovery + Bioenergy (low)* | 113 | 178 | 92 |
| *Higher Recovery + Bioenergy (high)* | 94 | 138 | 65 |
| *Portfolio (high)* | 34 | N/A | 35 |
| *Portfolio (low)* | 29 | N/A | 30 |


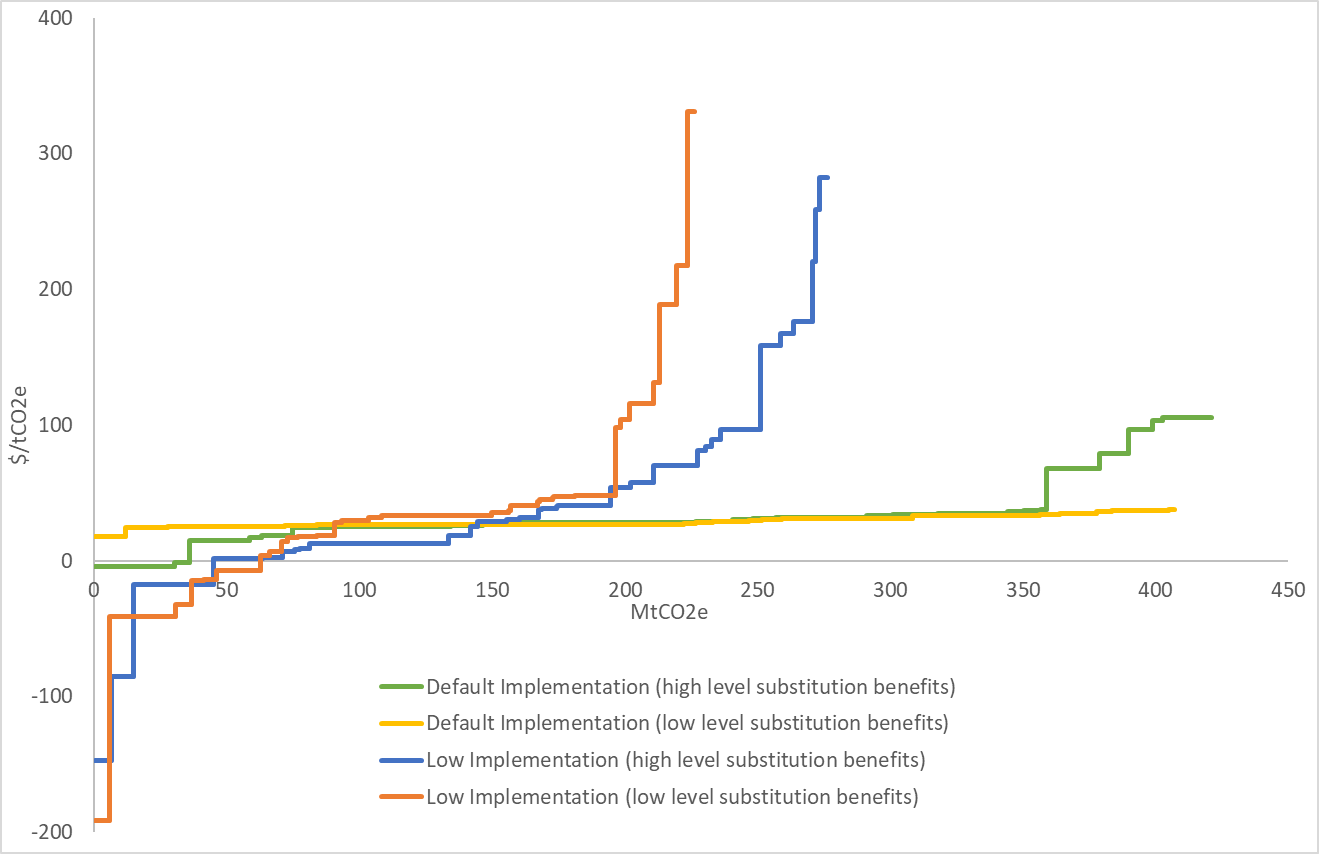


*Figure S9. Cost curves for domestic portfolios with default and low implementation levels and high and low substitution benefits, 2020-2070. The portfolios for the high implementation level were not included because the mitigation cost and socio-economic impact were not modeled for the high implementation level of the Harvest Less scenario.*

*Table S19. Socio-economic Impacts by scenario, implementation level and substitution benefit, 2020-2070.*

| Implementation Level | Scenario | Employment (direct) | Employment (total) | Annual Forest Sector GDP | Annual Provincial GDP | Annual Government Revenue |
| --- | --- | --- | --- | --- | --- | --- |
|  | (level of substitution benefits) | (full-time equivalent) | (full-time equivalent) | (2018$M/year) | (2018$M/year) | (2018$M/year) |
| Default | *Higher Recovery (low)* | 947 | 1,921 | 67.88 | 131.65 | 8.97 |
|  | *Higher Recovery (high)* | 993 | 2,015 | 71.02 | 137.77 | 9.39 |
|  | *Harvest Less (low / high)* | -1,167 | -2,362 | -102.44 | -196.49 | -13.66 |
|  | *Bioenergy (high)* | 1,040 | 1,880 | 314.50 | 362.95 | 47.67 |
|  | *Bioenergy (low)* | 1,040 | 1,880 | 298.81 | 344.85 | 45.29 |
|  | *Higher Recovery + Bioenergy (low)* | 1,649 | 3,130 | 319.37 | 404.73 | 47.50 |
|  | *Higher Recovery + Bioenergy (high)* | 1,760 | 3,339 | 340.95 | 431.79 | 50.72 |
|  | *Restricted Harvest (low / high)* | -945 | -1,912 | -85.75 | -164.42 | -11.43 |
|  | *LLP (low / high)* | 333 | 351 | -45.74 | -103.30 | -9.17 |
|  | *Domestic portfolio (low)* | -1,019 | -2,343 | -155.95 | -312.52 | -23.44 |
|  | *Domestic Portfolio (high)* | -177 | -714 | -28.62 | -135.26 | -5.03 |
| Low | *Higher Recovery (low)* | 649 | 1,317 | 47.64 | 92.24 | 6.30 |
|  | *Higher Recovery (high)* | 681 | 1,381 | 49.79 | 96.44 | 6.59 |
|  | *Harvest Less (low / high)* | -231 | -467 | -20.37 | -39.08 | -2.72 |
|  | *Bioenergy (high)* | 851 | 1,538 | 251.93 | 290.75 | 38.18 |
|  | *Bioenergy (low)* | 792 | 1,431 | 240.31 | 277.34 | 36.42 |
|  | *Higher Recovery + Bioenergy (low)* | 1,222 | 2,303 | 265.87 | 329.43 | 39.73 |
|  | *Higher Recovery + Bioenergy (high)* | 1,247 | 2,349 | 254.18 | 316.34 | 37.95 |
|  | *Restricted Harvest (low / high)* | -414 | -838 | -39.23 | -75.35 | -5.23 |
|  | *LLP (low / high)* | 182 | 192 | -26.23 | -59.14 | -5.23 |
|  | *Domestic portfolio (low)* | 565 | 826 | 79.48 | 44.26 | 10.78 |
|  | *Domestic Portfolio (high)* | 1,001 | 1,699 | 157.81 | 157.27 | 22.33 |
| High | *Higher Recovery (low)* | 1,359 | 2,757 | 95.80 | 185.99 | 12.65 |
|  | *Higher Recovery (high)* | 1,463 | 2,967 | 102.86 | 199.76 | 13.58 |
|  | *Harvest Less (low / high)* | - | - | - | - | - |
|  | *Bioenergy (high)* | 1,233 | 2,226 | 348.95 | 402.72 | 52.89 |
|  | *Bioenergy (low)* | 1,224 | 2,211 | 345.24 | 398.44 | 52.33 |
|  | *Higher Recovery + Bioenergy (low)* | 2,232 | 4,271 | 409.39 | 530.31 | 60.60 |
|  | *Higher Recovery + Bioenergy (high)* | 2,379 | 4,551 | 442.75 | 572.27 | 65.57 |
|  | *Restricted Harvest (low / high)* | -1,631 | -3,297 | -148.42 | -283.91 | -19.82 |
|  | *LLP (low / high)* | 485 | 509 | -65.25 | -147.45 | -13.11 |
|  | *Domestic portfolio (low)* | - | - | - | - | - |
|  | *Domestic Portfolio (high)* | - | - | - | - | - |

References

Arena, U., Di Gregorio, F., and Santonastasi, M. 2010. A techno-economic comparison between two design configurations for a small scale, biomass-to-energy gasification based system. Chem. Eng. J. **162**(2): 580-590. doi:10.1016/j.cej.2010.05.067.

BC Ministry of Forests and BC Environment. 1995. Biodiversity guidebook. British Columbia Environment, ISBN 0772626197, 99 pgs.

BC MOECCS. 2015. BC 2015 Industrial Facility Greenhouse Gas Emissions, BC Ministry of Environment and Climate Change Strategy, <https://www2.gov.bc.ca/gov/content/environment/climate-change/data/ceei>, Accessed Nov. 28, 2017.

BC MOECCS. 2016. BC 2016 Community Energy and Emissions Inventory, BC Ministry of Environment and Climate Change Strategy. <https://www2.gov.bc.ca/gov/content/environment/climate-change/data/ceei>, Accessed Mar. 3, 2018.

Dunn, D.W. 2011. Opportunities for utilization of natural gas for electricity generation. Fekete Associates Inc. <http://www.yukonenergy.ca/media/site_documents/charrette/docs/papers/NATURAL_GAS_YEC_Background_Paper.pdf>. Accessed May 4, 2015

EIA. 2013a. Updated capital cost estimates for utility scale electricity generating plants. Energy Information Administration, US Department of Energy. [www.eia.gov/forecasts/capitalcost/pdf/updated_capcost.pdf](http://www.eia.gov/forecasts/capitalcost/pdf/updated_capcost.pdf), Accessed May 4, 2015.

EIA. 2013b. Waste fuels are a significant energy source for U.S. manufacturers. Energy Information Administration, US Department of Energy.

<http://www.eia.gov/todayinenergy/detail.cfm?id=13531>. Accessed May 4, 2015.

Franklin, J.F., Spies, T.A., Van Pelt, R., Carey, A.B., Thornburgh, D.A., Berg, D.R., Lindenmayer, D.B., Harmon, M.E., Keeton, W.S., and Shaw, D.C. 2002. Disturbances and structural development of natural forest ecosystems with silvicultural implications, using Douglas-fir forests as an example. For. Ecol. Manag. **155**(1-3): 399-423.

Friesen, C. 2013. BC Central Interior Initiative, Forestry and Fibre Working Group. FPInnovations.

Hamilton Home Products. 2015. Characteristics of a Winchester electric furnace. <https://www.hamiltonhomeproducts.com/product/winchester-multi-positional-air-handler-electric-furnace> Accessed May 4th, 2015.

Howard, C. and Smyth, C. 2018. Bioenergy Mitigation Potential: Refining Displaced Emissions from British Columbia’s Heat and Electricity Production. Canadian Forest Serivce, Pacific Forestry Centre.

IEA. 2010. Industrial Combustion Boilers. Energy Technology Systems Analysis Programme, technology brief, I01. International Energy Agency. [www.iea-etsap.org/web/e-techds/pdf/i01-ind_boilers-gs-ad-gct1.pdf](http://www.iea-etsap.org/web/e-techds/pdf/i01-ind_boilers-gs-ad-gct1.pdf). Acessed May 4^th^, 2015.

MacDonald, A.J., Bernardo, J., and Spencer, S. 2012. Assessment of Forest Feedstock (Biomass) for Campbell River. FPInnovations, Vancouver.

Manitoba Hydro. 2015. Current natural gas rates. [https://www.hydro.mb.ca/regulatory_affairs/energy_rates/natural_gas/current_rates.shtml Accessed May 4](https://www.hydro.mb.ca/regulatory_affairs/energy_rates/natural_gas/current_rates.shtml%20%20%20%20%20Accessed%20May%204), 2015.

# Metsaranta, J. M., Dymond, C., Kurz, W. A., Spittlehouse, D. L, 2011, Uncertainty of 21st

# century growing stocks and GHG balance of forests in British Columbia, Canada resulting from potential climate change impacts on ecosystem processes. For. Ecol. Manag. 262, 827-837.

NEB. 2014. Market snapshot: continuing high prices in the maritimes’ distinct natural gas market. National Energy Board. <http://www.neb-one.gc.ca/nrg/ntgrtd/mrkt/snpsht/2014/12-02mrtmngmrkt-eng.html>. Accessed May 4th, 2015.

NEB. 2017. National Energy Board Canada’s Energy Future 2017 – Energy Supply and Demand Projections to 2040. National Energy Board. [https://www.neb-one.gc.ca/nrg/ntgrtd/ftr/2017/index-eng.html Accessed Feb 26](https://www.neb-one.gc.ca/nrg/ntgrtd/ftr/2017/index-eng.html%20Accessed%20Feb%2026), 2018.

NRCAN. 2015. Fuel focus database. Natural Resources Canada. <http://www.nrcan.gc.ca/energy/fuel-prices/4593>. Accessed May 4th, 2015.

NRMCA. 2012. Ready mixed concrete industry data report (executive summary). National Ready Mixed Concrete Association, <https://www.nrmca.org/members/downloads/industrydatasurvey2012.pdf>. Accessed Sept. 30 2015.

Paradis, G. 2018. DEG: A disturbance event generator for GCBM, Internal report.

Pröll, T., Rauch, R., Aichernig, C., and Hofbauer, H. 2011. Fluidized Bed Steam Gasification of Solid Biomass - Performance Characteristics of an 8 MWth Combined Heat and Power Plant. International Journal of Chemical Reactor Engineering: **5**: A54. Available from http://www.bepress.com/ijcre/vol5/A54

Ralevic, P. 2013. Evaluating the greenhouse gas mitigation potential and cost-competitiveness of forest bioenergy systems in northeastern Ontario University of Toronto.

Ralevic, P., Ryans, M., and Cormier, D. 2010. Assessing forest biomass for bioenergy: Operational challenges and cost considerations. For. Chron. **86**(1): 43-50.

RECD, 2017, Remote Communities Energy Database (The Atlas of Canada):
<http://atlas.gc.ca/rced-bdece/en/index.html>, Accessed Mar. 5, 2018.

RETScreen International. 2015. RETScreen Project Database. <http://www.retscreen.net/ang/software_and_data.php>. Accessed Sept. 11^th^, 2015.

Reynolds, R., Volpe, S., and Cormier, D. 2012. Northwestern Ontario forest-origin feedstock supply analysis for a potential pellet production (Feedstock analysis only) FPInnovations, Pointe-Claire, QC.

Ryans, M. and Cormier, D. 2009. Opportunities and challenges to biomass harvesting: Operational perspective, FPInnovations - Feric Blandin Foundation – OMNR Tour, Thunder Bay, ON.

Smyth, C., Kurz, W.A., Rampley, G.J., Lemprière, T.C., and Schwab, O. 2017a. Climate change mitigation potential of local use of harvest residues for bioenergy in Canada. Global Change Biology Bioenergy **9**: 817–832. doi:doi:10.1111/gcbb.12387.

US EPA. 2013. Fact sheet: CHP as a boiler replacement opportunity. Environmental Protection Agency. <https://www.epa.gov/sites/production/files/2015-07/documents/fact_sheet_chp_as_a_boiler_replacement_opportunity.pdf>. Accessed May 4th, 2015.

USDC. 2011. Architectural manual: expected useful life table. US Department of Commerce. http://[www.commerce.wa.gov/Documents/Expected-Useful-Life.pdf](http://www.commerce.wa.gov/Documents/Expected-Useful-Life.pdf). Accessed May 4th, 2015.

Wells, R.W., Lertzman, K.P., and Saunders, S.C. 1998. Old-growth definitions for the forests of British Columbia, Canada. Nat. Areas J. **18**(4): 279-292.

Wong, C., Dorner, B., and Sandmann, H. 2003. Estimating historical variability of natural

disturbances in British Columbia. Ministry of Sustainable Resource Management,

Resource Planning Branch. ISBN 0-7726-5060-8.

Wood, S.R. and Rowley, P.N. 2011. A techno-economic analysis of small-scale, biomass-fuelled combined heat and power for community housing. Biomass Bioenergy **35**(9): 3849-3858. doi:10.1016/j.biombioe.2011.04.040.
